# LNGFR^+^THY-1^+^VCAM-1^hi+^ Cells Reveal Functionally Distinct Subpopulations in Mesenchymal Stem Cells

**DOI:** 10.1016/j.stemcr.2013.06.001

**Published:** 2013-07-11

**Authors:** Yo Mabuchi, Satoru Morikawa, Seiko Harada, Kunimichi Niibe, Sadafumi Suzuki, Francois Renault-Mihara, Diarmaid D. Houlihan, Chihiro Akazawa, Hideyuki Okano, Yumi Matsuzaki

**Affiliations:** 1 Department of Physiology, Keio University School of Medicine, Shinjuku-ku, Tokyo 160-8582, Japan; 2 Department of Dentistry and Oral Surgery, Keio University School of Medicine, Shinjuku-ku, Tokyo 160-8582, Japan; 3 Department of Biochemistry and Biophysics, Graduate School of Health Care Sciences, Tokyo Medical and Dental University, Bunkyo-ku, Tokyo 113-8510, Japan; 4 Centre for Liver Research, NIHR Biomedical Research Unit, University of Birmingham, Birmingham B15 2TT, UK; 5 Institute of Medical Science, Tokyo Medical University, Shinjuku-ku, Tokyo 160-8402, Japan

## Abstract

Human mesenchymal stem cells (hMSCs), which conventionally are isolated based on their adherence to plastic, are heterogeneous and have poor growth and differentiation, limiting our ability to investigate their intrinsic characteristics. We report an improved prospective clonal isolation technique and reveal that the combination of three cell-surface markers (LNGFR, THY-1, and VCAM-1) allows for the selection of highly enriched clonogenic cells (one out of three isolated cells). Clonal characterization of LNGFR^+^THY-1^+^ cells demonstrated cellular heterogeneity among the clones. Rapidly expanding clones (RECs) exhibited robust multilineage differentiation and self-renewal potency, whereas the other clones tended to acquire cellular senescence via *P16INK4a* and exhibited frequent genomic errors. Furthermore, RECs exhibited unique expression of VCAM-1 and higher cellular motility compared with the other clones. The combination marker LNGFR^+^THY-1^+^VCAM-1^hi+^ (LTV) can be used selectively to isolate the most potent and genetically stable MSCs.

## Highlights

• The LNGFR^+^THY-1^+^ population is significantly enriched for CFU-Fs in human bone marrow
• Rapidly expanding clones (RECs) exhibited stem-like characteristics
• Expression of VCAM-1 correlated with proliferation and migration ability
• The combination marker LNGFR^+^THY-1^+^VCAM-1^hi+^ is useful for isolating multipotent MSCs

## Graphical Abstract

**Figure:**
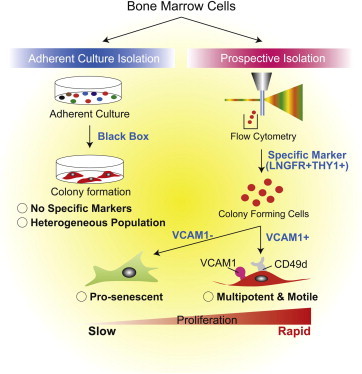

## Introduction

Mesenchymal stem/stromal cells (MSCs) are defined as nonhematopoietic, plastic-adherent, self-renewing cells that are capable of in vitro trilineage differentiation into fat, bone, and cartilage (Pittenger et al., 1999). Additional plasticity of MSCs has been suggested by experiments demonstrating their in vitro differentiation into myocytes, neuron-like cells, and hepatocytes (Drost et al., 2009; Galvin and Jones, 2002; Tao et al., 2009). Despite these data, the term “MSCs” has been controversial, as a definitive demonstration of their “stemness” by single-cell isolation and in vivo serial transplantation experiments has been lacking (Bianco et al., 2013). These multipotent cells are found in various fetal and adult human tissues, including bone marrow (BM), umbilical cord blood (UCB), liver, and term placenta (Battula et al., 2007; Erices et al., 2000; Yen et al., 2005; Zvaifler et al., 2000). MSCs are multipotent and have low immunogenicity, and therefore are considered as potential candidates for a variety of clinical applications (Jung et al., 2012; Stappenbeck and Miyoshi, 2009), including cartilage reconstitution and the treatment of rheumatoid arthritis, acute osteochondral fractures, spinal disk injuries, and inherited diseases such as osteogenesis imperfecta (Guillot et al., 2008). However, to date, these cells have been poorly characterized, which raises significant concerns because human trials using MSCs are currently under way.

MSCs can be retrospectively identified based on their ability to form colony-forming unit fibroblasts (CFU-Fs) in vitro (Friedenstein et al., 1974). Traditionally, the isolation of MSCs from unfractionated whole BM (WBM) has relied on their adherence to plastic dishes. This technique gives rise to heterogeneous cell populations that frequently are contaminated with osteoblasts and/or osteoprogenitor cells, fat cells, reticular cells, macrophages, endothelial cells, and hematopoietic cells (Pittenger et al., 1999). Prolonged culture is often required to remove these contaminants and obtain a reasonably pure population of MSCs. However, during this process, the differentiation, proliferation, and migration potency of the MSCs gradually diminishes as the cells acquire a more mature phenotype (Kim et al., 2009; Rombouts and Ploemacher, 2003). In an effort to overcome these problems, investigators have made an intense effort to identify reliable MSC surface markers that could facilitate the prospective isolation of colony-initiating cells.

Various surface markers, including CD49a, CD73, CD105, CD106 (VCAM-1), CD140b, CD146, CD271 (LNGFR), MSCA-1, and STRO-1, have been used alone or in combination to isolate human MSCs (hMSCs) (Aslan et al., 2006; Battula et al., 2009; Boiret et al., 2005; Bühring et al., 2007; Gronthos et al., 2003; Quirici et al., 2002; Sacchetti et al., 2007). CD49a, CD73, CD140b, and CD146 are widely expressed in stromal cells (e.g., pericytes and reticular cells) and thus are not unique to MSCs. STRO-1 is a popular MSC marker and is often used in combination with VCAM-1 for MSC isolation. However, these markers are also found on some hematopoietic cells, and additional markers, including CD45 and Glycophorin A (GPA), are required to exclude contaminating cells (Gronthos et al., 2003; Simmons and Torok-Storb, 1991). Therefore, the identification of a combination of cell surface markers specific to hMSCs has remained an important prerequisite for the repeated isolation of purified multipotent MSC fractions.

In the present study, we performed a comprehensive screening of putative surface markers to select the most useful ones for prospectively identifying a pure MSC population in human BM. We describe a significantly improved method that enables the simple and reliable prospective isolation of MSCs based on their expression of LNGFR, THY-1, and VCAM-1.

## Results

### Identification of MSC Markers

**Figure 1:**
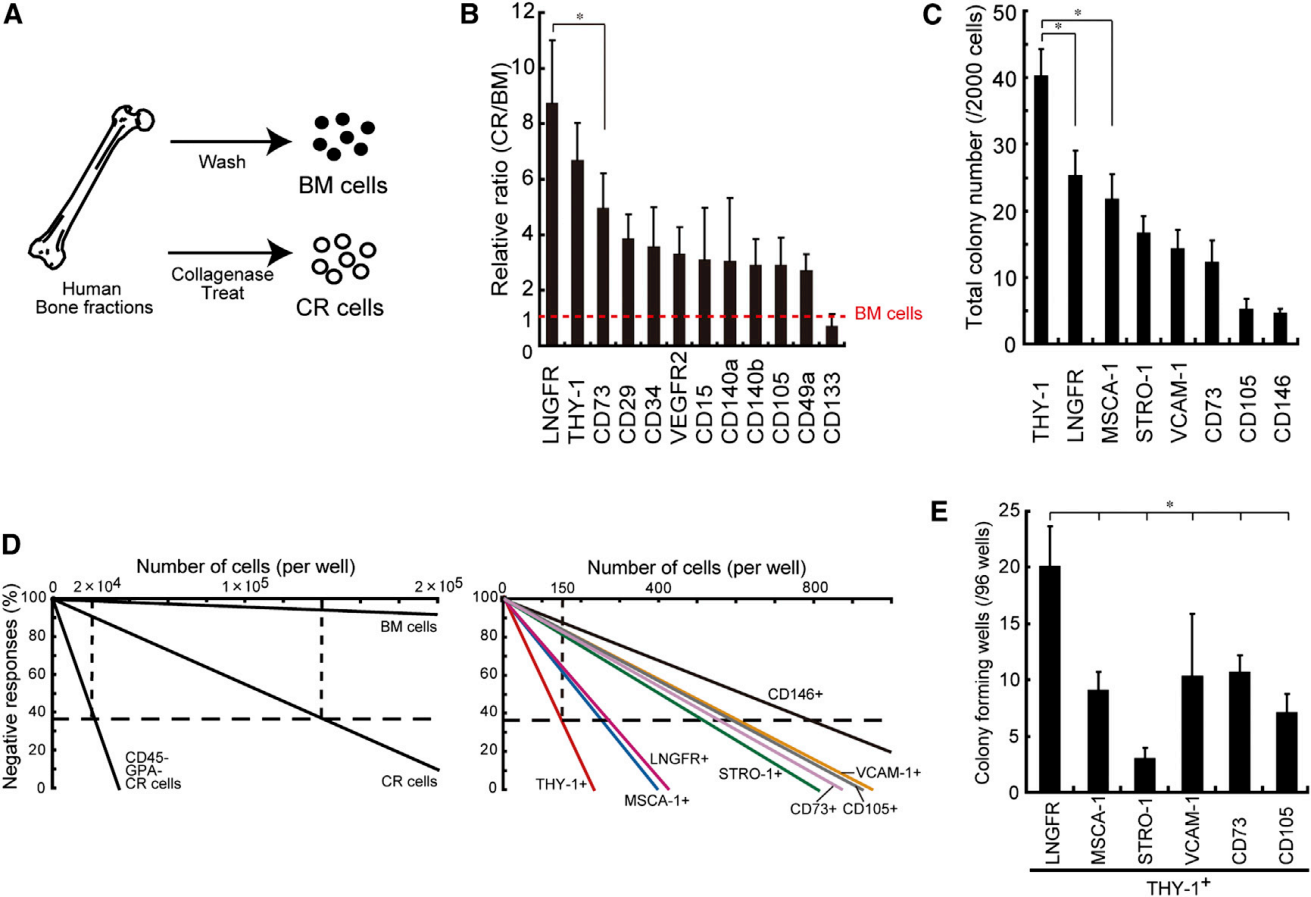
Screening of Putative Surface Markers for Prospective Identification of hMSCs (A) Human bone fragments were washed with PBS (BM cells) or treated with collagenase (CR cells). (B) Expression ratio of the indicated surface markers in the CD45^−^ and GPA^−^ cell populations. The data are shown as the ratio of the expression of a given marker on CR cells versus BM cells (mean ± SEM, n = 5; ^∗^p < 0.1). (C) Colony-forming assay of a single marker population (2,000 cells) in CR cells at 14 days (mean ± SEM, n = 6 per group; ^∗^p < 0.05). (D) Assay measuring the negative linear relationship between the numbers of seeded cells (BM, CR, and CD45^−^GPA^−^ CR cells) and sorted cells, using a surface marker in CR cells. (E) Clonogenic assay of single cells seeded into 96-well plates and cultured for 14 days (BM-MNC POIETICS were used in this assay; mean ± SEM, n = 3; ^∗^p < 0.05). See also Figure S1.

We isolated fresh human BM cells using either the traditional method of flushing the BM or collagenase digestion of crushed bone (collagenase-released [CR] cells), as previously described for a murine MSC isolation procedure (Houlihan et al., 2012; Morikawa et al., 2009; Figure 1A). We initially examined the CFU-F potential of these two cell types by plating 10^3^, 10^4^, or 10^5^ cells, and counting the wells with formed colonies. After a 2-week culture period, we found that the CFU-F frequency was far greater with CR cells than with BM cells using the standard isolation method (BM: 3.3% ± 3.3%, 7.4% ± 7.4%, 0% ± 0%; CR: 24.8% ± 5.2%, 35.6% ± 15.6%, 77.8% ± 22.2%; Figure S1A available online). Based on these findings, we concluded that collagenase treatment and depletion of hematopoietic cells increases the frequency of CFU-F formation. This prompted us to search for putative cell-surface markers specific to this potent subpopulation. To that end, we used flow cytometry to screen >100 potential markers on both BM and CR cells. We specifically looked for “rare” (<0.1%) surface markers whose frequency in the nonhematopoietic fraction increased following collagenase treatment. The frequencies of cells expressing some known MSC markers increased among CR cells, and in particular, cells expressing LNGFR and THY-1 increased significantly in the CR fraction compared with flushed BM (CR/BM ratio: LNGFR: 8.75 ± 2.3; THY-1: 6.70 ± 1.3; Figure 1B).

To assess the purification efficiency of these potential markers (THY-1 and LNGFR) and compare them with previously validated MSC markers (MSCA-1, STRO-1, VCAM-1, CD73, CD105, and CD146), we performed CFU-F assays using CR cells based on their expression of each marker. The THY-1^+^ cells showed the greatest CFU-F frequency among the fractions tested (Figure 1C). A limiting dilution assay measuring the relationship between seeding density and colony-forming efficiency also indicated that THY-1^+^ cells had the highest clonogenic potential (1 CFU-F per 150 cells seeded; Figure 1D). To determine whether a particular combination of surface markers could select for a more potent population of hMSCs, we examined cells that were positive for each selected marker (LNGFR, MSCA-1, STRO-1, VCAM-1, CD73, or CD105), combined with THY-1 expression (Figure 1E). Additionally, previously reported MSC marker combinations, including LNGFR^+^CD140a^+^ and LNGFR^+^CD146^+^, were tested (Figure S1B). Single-cell assays demonstrated that LNGFR^+^THY-1^+^ cells had the highest CFU-F potential (Figures 1E and S1B). Collectively, the LNGFR^+^THY-1^+^ cells demonstrated a CFU-F frequency that was ∼200,000 times higher than observed for unfractionated BM cells (LNGFR^+^THY-1^+^ cells versus BM cells: 1 per 5–6 cells seeded versus 1 per 1.2 × 10^6^ cells seeded; Figures 1D and 1E). In addition, flow-cytometric analysis showed that the LNGFR^+^THY-1^+^ population contained the smallest percentage of hematopoietic cells (CD45^+^: 1.01%) and red blood cells (GPA^+^: 0.85%) among the combinations tested (Figure S1C). We confirmed by immunocytochemistry that the LNGFR^+^THY-1^+^ population was not contaminated by endothelial cells (CD31-staining negative) or osteogenic cells (osteocalcin-staining negative; Figure S1D). Therefore, two-color immunostaining with THY-1 and LNGFR is a reliable method for isolating MSCs from BM with minimal contamination from hematopoietic cells.

### hMSCs Exist Only in the LNGFR^+^THY-1^+^ Population

**Figure 2:**
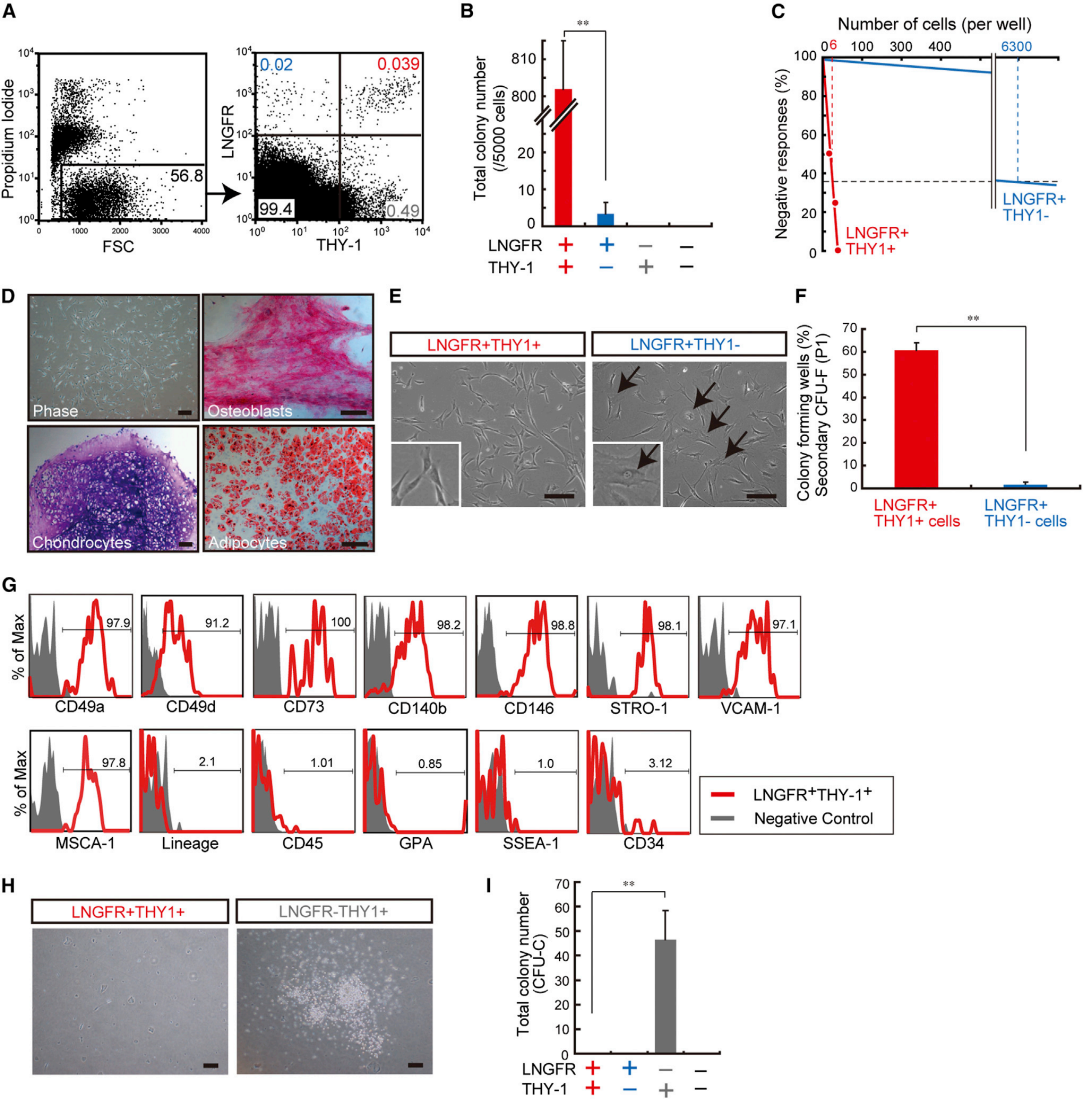
The LNGFR^+^THY-1^+^ Population Is Significantly Enriched for CFU-Fs with Potent Differentiation Potential (A) Representative flow-cytometric profiles of human BM stained for LNGFR and THY-1. (B) Numbers of CFU-Fs detected 14 days after plating 5,000 cells from each of the following groups: LNGFR^+^THY-1^+^, +/−, −/+, and −/− cells (mean ± SEM, n = 12 per group; ^∗∗^p < 0.01). (C) Assay of the negative linear relationship between the numbers of seeded LNGFR^+^THY-1^+^ and LNGFR^+^THY-1^−^ cells. (D) Phase-contrast micrographs of a colony of LNGFR^+^THY-1^+^ cells (phase), showing the potential of a LNGFR^+^THY-1^+^ colony to differentiate into osteoblasts, chondrocytes, and adipocytes. Scale bar = 100 μm. (E) Phase-contrast micrographs of LNGFR^+^THY-1^+^ and LNGFR^+^THY-1^−^ colonies (passage 1). Arrows point to cells with larger amounts of cytoplasm. Scale bar = 100 μm. (F) Secondary CFU-Fs assays were performed with single cells sorted from LNGFR^+^THY-1^+^ and LNGFR^+^THY-1^−^ colonies after one passage (mean ± SEM, n = 3; ^∗∗^p < 0.01). (G) Flow-cytometric analysis of surface markers on LNGFR^+^THY-1^+^ cells, showing the percentage of cells that express the antigen (red line) versus a matched isotype control (gray). Lineage cocktail: CD3, CD14, CD16, CD19, CD20, and CD56. (H) Phase-contrast image of cultures of LNGFR^+^THY-1^+^ and LNGFR^−^THY-1^+^ cells after 14 days of culture in MethoCult medium (CFU-C assay). Scale bar = 100 μm. (I) Total numbers of CFU-Cs counted on day 14 (mean ± SEM, n = 3; ^∗∗^p < 0.01). BM-MNC POIETICS were used for all experiments in this figure. See also Figures S2 and S3.

Two-color staining for LNGFR and THY-1 of WBM revealed the presence of four distinct subpopulations (LNGFR^+^THY-1^+^, +/−, −/+, and −/−; Figure 2A). We isolated cells from each subpopulation and performed several in vitro assays to determine their characteristics. The traditional CFU-F and limiting dilution assays indicated that the number of colony-forming cells arising from each subpopulation was highest in the LNGFR^+^THY-1^+^ subpopulation, with a frequency of one in six (Figures 2B and 2C). When the in vitro mesenchymal lineage differentiation potential was investigated, CFU-Fs from LNGFR^+^THY-1^+^ cells robustly differentiated into adipocytes, chondrocytes, and osteoblasts (Figure 2D). In contrast, cells in the other subpopulations had extremely low CFU-Fs (1/6,300 in LNGFR^+^THY-1^−^) or no CFU-Fs (LNGFR^−^THY-1^−^ and LNGFR^−^THY-1^+^; Figures 2B and 2C). The few colonies that formed from LNGFR^+^THY-1^−^ cells appeared to have nonfibroblastic shapes (Figure 2E, arrows) and rarely formed secondary CFU-Fs when reseeded, although 60% of CFU-Fs from LNGFR^+^THY-1^+^ cells produced secondary colonies under the same conditions (Figure 2F). These results suggested that CFU-Fs in the LNGFR^+^THY-1^−^ population were not hMSCs. Multipotent and self-renewing MSCs were only present in the LNGFR^+^THY-1^+^ fraction.

Multicolor flow-cytometric analysis indicated that naive LNGFR^+^THY-1^+^ cells uniformly expressed CD49a, CD49d, CD73, CD140b, CD146, STRO-1 VCAM-1, and MSCA-1 (Figure 2G). In contrast, almost all of the cells were negative for hematopoietic lineage markers (CD3, CD14, CD16, CD19, CD20, CD45, CD56, and GPA), an embryonic stem cell marker (SSEA-1), and an endothelial/hematopoietic stem cell (HSC) marker (CD34; Figure 2G). To confirm that the LNGFR^+^THY-1^+^ population was devoid of hematopoietic progenitor cells, we performed a colony-forming units in culture (CFU-C) assay to detect hematopoietic progenitor cells. The results showed that LNGFR^+^THY-1^+^ cells produced no CFU-Cs (Figure 2H). However, LNGFR^−^THY-1^+^ cells, which exhibited no CFU-F potential, formed >40 CFU-Cs per 5,000 cells seeded under the same culture conditions (Figure 2I). These data strongly suggested that LNGFR and THY-1 could be used for the prospective identification of a rare and potent population of hMSCs.

We looked for the presence of LNGFR^+^THY-1^+^ cells in a variety of other human tissues that contain MSC-like cells, including placenta (amnion, chorion, and decidua), fat, peripheral blood (PB, with or without G-CSF mobilization), and UCB (Lee et al., 2004; Nagase et al., 2009; Zimmerlin et al., 2010; Zvaifler et al., 2000). LNGFR^+^THY-1^+^ cells were detected in the decidua but not the amnion or chorion of the placenta (Figure S2A). Decidual LNGFR^+^THY-1^+^ cells exhibited a high CFU-F potential equivalent to that of BM-derived LNGFR^+^THY-1^+^ cells (Figure S2B). Interestingly, both the LNGFR^+^THY-1^−^ and LNGFR^−^THY-1^−^ fractions, derived from adipose tissue, generated CFU-Fs, albeit less frequently than LNGFR^+^THY-1^+^ cells (Figures S2C and S2D). We were unable to detect LNGFR^+^THY-1^+^ cells among 10^6^ PB or UCB cells; however, a small number of LNGFR^+^THY-1^+^ cells that had clonogenic potential were present in G-CSF-mobilized PB (G-CSF mPB; Figures S2E and S2F).

In a previous study (Morikawa et al., 2009), we reported that, after undergoing ex vivo expansion, intravenously injected MSCs were trapped largely in the lung and did not migrate to the BM compartment. To evaluate homing of cultured and naive LNGFR^+^THY-1^+^ cells to bone in vivo, we transplanted the cells into immunodeficient mice (Figure S3A). Mice injected with 2 × 10^5^ to 1 nbsp;× 10^6^ LNGFR^+^THY-1^+^ cells expanded in culture, died from pulmonary embolism within a week, and no human cells could be identified in the recipients’ BM. In contrast, 3 months after the transplantation of 1 × 10^4^ freshly isolated LNGFR^+^THY-1^+^ cells, we were able to collect human-derived cells from the recipients’ BM using species-specific antibodies. We performed immunohistochemistry after LNGFR^+^THY-1^+^ cell transplantation and observed the transplant human cells in mouse BM. Human THY-1-positive cells were detected in lung, skin, and subcutaneous tissue; however, expression of α-SMA and alkaline phosphatase (ALP) was not detected (Figure S3B). To test whether these surviving human cells retained their stem cell properties, we sorted human THY-1^+^ cells and performed several in vitro assays (Figure S3C). Following expansion in vitro, the sorted human THY-1^+^ cells were capable of colony formation and multilineage differentiation into bone, cartilage, and fat cells (Figure S3D). These data demonstrated that freshly isolated human LNGFR^+^THY-1^+^ cells could home to the BM and retain their proliferative and multilineage differentiation potential in murine recipients, although the engraftment efficiency was low.

### Defining Subpopulations of CFU-Fs Using Single-Cell Assays

**Figure 3:**
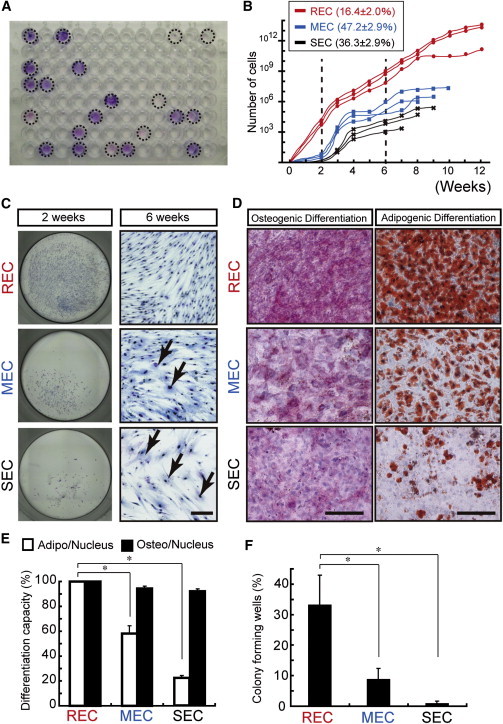
Clonal Assay of LNGFR^+^THY-1^+^ Cells (A) Single-cell assay in a LNGFR^+^THY-1^+^ population. Representative image of a typical 96-well culture plate (day 21 after initial seeding) shows colony-forming wells. (B) Single-cell sorting and in vitro expansion reveals three distinct MSC subpopulations of rapidly, moderately, or slowly expanding MSC clones (RECs, MECs, and SECs, respectively). Growth curves of representative RECs, MECs, and SECs, and the percentage of each colony type present are shown. (C) Phase-contrast micrograph showing the cellular morphologies of RECs, MECs, and SECs. The arrows point to larger MECs and SECs with larger nuclei. Scale bar = 100 μm. (D) Differentiation of each colony type into adipocytes and osteoblasts. Scale bar = 100 μm. (E) Quantitative analysis of the differentiation capacity of each colony type (differentiation cells / nuclei; mean ± SEM, n = 6; ^∗^p < 0.05). (F) Average number of secondary CFU-Fs in wells for each cell type at 6 weeks (mean ± SEM, n = 3; ^∗^p < 0.05). BM-MNC POIETICS were used for all experiments in this figure. See also Table S1.

To further define the differentiation and proliferation potential of LNGFR^+^THY-1^+^ cells, we monitored colony formation after single-cell seeding into a 96-well culture dish (Figure 3A). Interestingly, analysis of their growth kinetics revealed that single LNGFR^+^THY-1^+^-derived CFU-Fs could be divided into three distinct subgroups: rapidly expanding MSC clones (RECs: 16.4% ± 2.0%) that yielded >10^5^ cells by day 14, moderately expanding MSC clones (MECs: 47.2% ± 2.9%) that took up to 21 days to yield 10^5^ cells, and slowly expanding MSC clones that failed to generate 10^5^ cells after 1 month of culture (SECs: 36.3% ± 2.9%; Figure 3B). The subgroups had different cellular morphologies. RECs were uniformly small and spindle-shaped, whereas MECs and SECs were polymorphic and contained cells with larger nuclei and enlarged cytoplasm (Figure 3C, arrows in phase images). The heterogeneity among these groups is also reflected in the different cell size of RECs compared with MECs and SECs (Figure 3C). When the adipogenic and osteogenic differentiation efficiencies of each subgroup were compared, there were clearly more adipogenic cells in RECs than in MECs or SECs (REC: 99.3% ± 0.33%; MEC: 58.1% ± 6.26%; SEC: 22.2% ± 1.87%; Figures 3D and 3E). In addition, RECs exhibited the greatest secondary colony-forming efficiency (RECs: 33.3% ± 9.6%; MECs: 8.9% ± 3.5%; SECs: 1.0% ± 0.58%) after the single-cell sorting of each subgroup (Figure 3F). We established individual MSC clones derived from single LNGFR^+^THY-1^+^ cells (n = 145; Table S1). All RECs were multipotent and capable of differentiating into either all three lineages (adipogenic, chondrogenic, and osteogenic [37/46: 80%]) or bilineage cells (9/46: 20%). Although some MECs/SECs were unable to differentiate (6/48: 13% and 7/51: 14%, respectively), >60% of MECs/SECs showed either tri- or bilineage differentiation potential (38/48: 79% of MECs and 32/51: 62% of SECs), indicating that most of the CFU-Fs derived from LNGFR^+^THY-1^+^ cells were multipotent. These data suggested that there is a functional hierarchy among CFU-Fs derived from clonal LNGFR^+^THY-1^+^ cells, with RECs exhibiting the most potent stem-like characteristics.

### Cultured MSCs Undergo Cellular Senescence via *P16INK4a*

**Figure 4:**
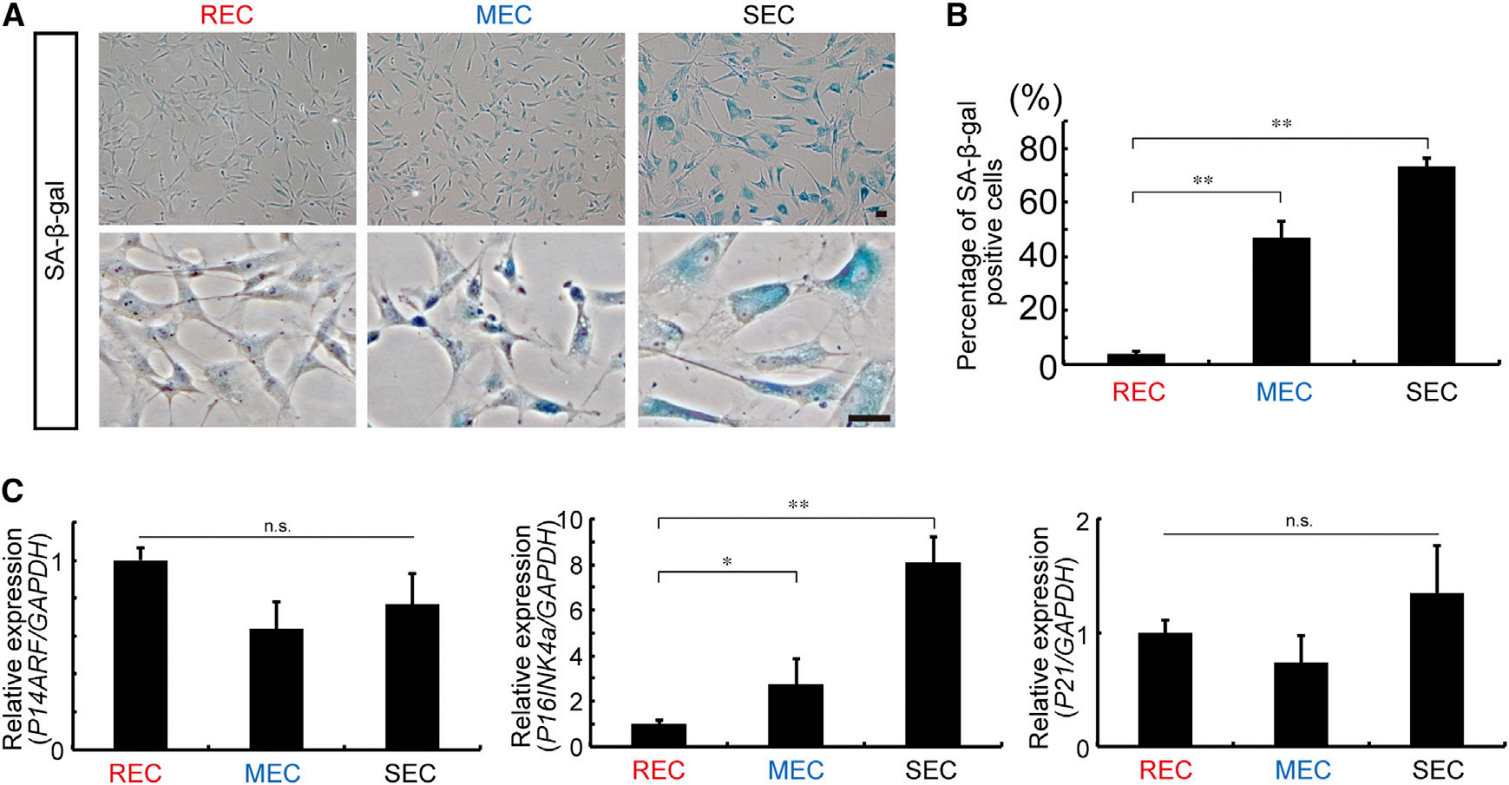
Heterogeneity in the MSC Compartment (A) SA-β-gal assay of RECs, MECs, and SECs cultured in a glass chamber (at 6 weeks). Scale bar = 50 μm. (B) Quantitative analysis of SA-β-gal^+^ cells in RECs, MECs, and SECs (mean ± SEM, n = 3 per group; ^∗∗^p < 0.01). (C) Relative expression of *P14ARF*, *P16INK4a*, and *P21* in 6-week cultured cells by real-time RT-PCR (mean ± SEM, n = 3; ^∗^p < 0.1, ^∗∗^p < 0.01; n.s., not significant). See also Figure S4 and Table S2.

We observed a greater increase in senescence-associated beta-galactosidase (SA-β-gal)-positive cells in MECs and SECs compared with RECs, demonstrating that MEC/SEC cells undergo cellular senescence (Figures 4A and 4B). To assess the genomic stability of RECs, MECs, and SECs, we performed array-based comparative genomic hybridization (aCGH) to investigate aneuploidy and genomic abnormalities (Ben-David et al., 2011; Spits et al., 2008). We found that MECs and SECs accumulated nonoverlapping copy-number variations (CNVs), indicating de novo genomic DNA abnormalities, although no errors were identified in any of the RECs studied (Table S2). Since DNA damage induces premature senescence via cell-cycle regulatory proteins in many cell types (Galderisi et al., 2009), we measured the gene expression of the tumor suppressor *P14ARF* and the cdk inhibitors *P16INK4a* and *P21* in each group. A significant increase in *P16INK4a* expression was observed in MECs and SECs compared with RECs, in contrast to *P21* and *P14ARF* expression (Figure 4C). Therefore, cellular senescence in MECs and SECs might be triggered by de novo genomic DNA abnormalities and increased *P16INK4a* expression.

Notably, a dose-response curve plotting the CFU-F number against the number of LNGFR^+^THY-1^+^ cells plated per well did not show a linear relationship. Instead, we obtained fewer than expected CFU-Fs from the higher cell concentrations (Figure S4A). In addition, we observed different growth kinetics with single REC, LNGFR^+^THY-1^+^ cells (1,000 cells), and WBM cells (1 × 10^6^). Specifically, single RECs continued to grow without senescence, generating >10^12^ cells after 10 weeks, whereas LNGFR^+^THY-1^+^ cells stopped proliferating after 6 weeks and yielded significantly fewer cells (∼10^10^ cells; Figure S4B). Logically, 1,000 LNGFR^+^THY-1^+^ cells should contain ∼170 CFU-Fs, with ∼30 comprised of RECs, ∼90 comprised of MECs, and ∼50 comprised of SECs. However, the total number of LNGFR^+^THY-1^+^ growth cells was much smaller than we anticipated. To analyze this phenomenon in detail and exclude the possibility of cellular overcrowding, we performed a coculture assay using RECs and SECs at low cell numbers (Figure S4C). Following 4 weeks in coculture, REC proliferation was markedly reduced in the presence of SECs (Figures S4D and S4E). These results suggest that the SEC-mediated inhibition of REC proliferation is independent of cell density. Therefore, the direct isolation of RECs should be the most effective way to obtain highly pure MSCs from mixed CFU-Fs.

### VCAM-1 and CD49d Are Specifically Expressed in RECs

Isolation of RECs is difficult because it requires single-cell sorting of LNGFR^+^THY-1^+^ cells followed by a minimum of 2 weeks in culture. Given the characteristics of RECs, which make them attractive for therapeutic applications, we attempted to identify REC-specific markers. “Index sorting” enabled us to identify cells in the LNGFR^+^THY-1^+^ fraction that gave rise to RECs. There was a higher concentration of RECs in the LNGFR and THY-1 bright fraction (66.6% in RECs); however, MECs and SECs were also present in the same region (51.4% in MECs, 45.0% in SECs; Figures S5A–S5C). Similarly, measurements of cell size (forward scatter) and complexity (side scatter) failed to distinguish among the groups (Figure S5D).

**Figure 5:**
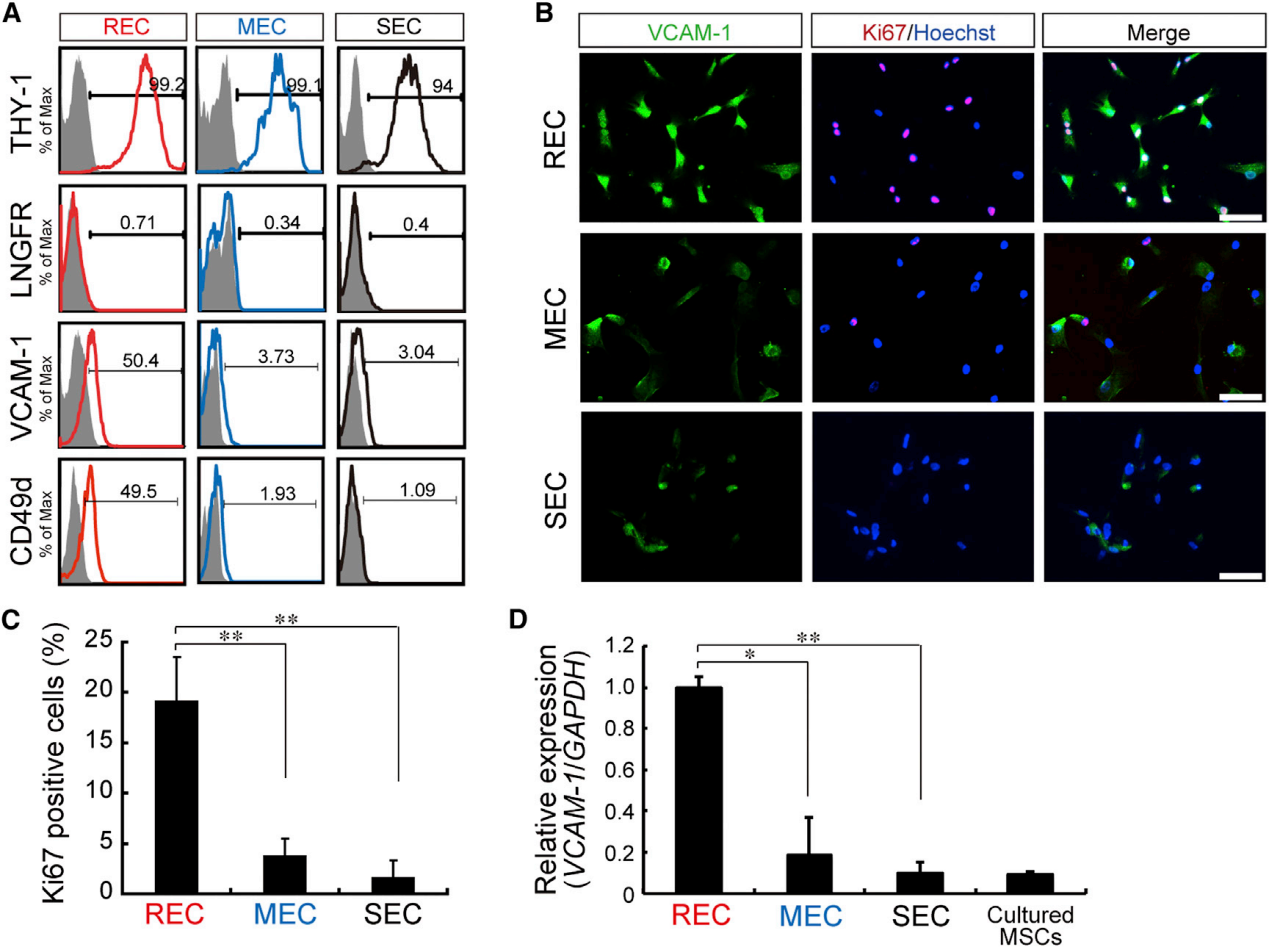
VCAM-1 Identifies Rapidly Dividing MSCs (A) Expression of the indicated surface markers in RECs, MECs, and SECs (6 weeks in culture). Shown is the percentage of cells that express the antigen (line) versus a matched isotype control (gray). (B) Immunocytochemistry of MSC subpopulation with VCAM-1 (green), Ki67 (red), and nuclei (blue). Scale bar = 100 μm. (C) Quantitative analysis of Ki67^+^ cells in RECs, MECs, and SECs (mean ± SEM, n = 3 per group; ^∗∗^p < 0.01). (D) Relative expression of *VCAM-1* marker in cultured cells (6 weeks) by real-time RT-PCR (mean ± SEM, n = 3; ^∗^p < 0.05, ^∗∗^p < 0.01). See also Figure S5.

The differential expression of various cell-surface molecules was explored further in each of the three cell types (Figures 5A and S5E). The observation of moderate VCAM-1 and CD49d (Integrin α4) expression on RECs, in contrast to their absence on MECs and SECs, was particularly noteworthy (Figure 5A). VCAM-1 and Ki67 immunohistological staining indicated a higher proliferation in RECs (19%) compared with MECs or SECs (<5%; Figures 5B and 5C). Quantitative PCR analysis confirmed a significantly higher expression of *VCAM-1* in RECs compared with MECs and SECs (Figure 5D).

### Cell Motility Is Mediated via VCAM-1 in RECs

**Figure 6:**
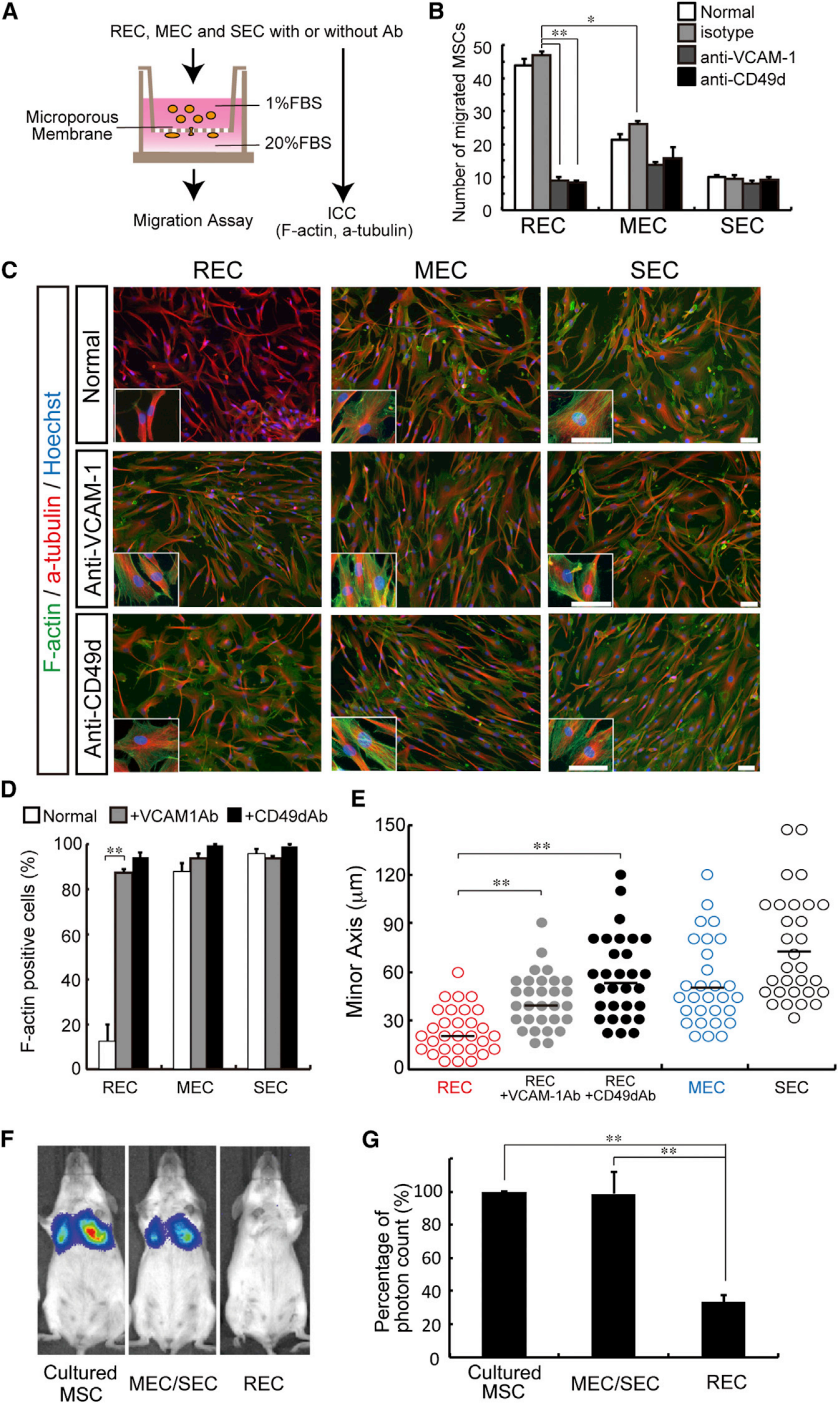
Expression of VCAM-1 and CD49d Correlated with the Migration Ability of MSCs (A) Migration assay in vitro. A total of 1.5 × 10^4^ cells (RECs, MECs, and SECs) were resuspended in DMEM containing 1% FBS and 10 μg/ml aphidicolin, and allowed to migrate toward the culture medium supplemented with 20% FBS in the lower chamber. (B) Migration assay of RECs, MECs, and SECs with or without blocking antibody (“Normal” indicates no antibody treatment). The cells were incubated with each antibody (isotype control [mouse IgG1], anti-VCAM-1 [51-10C9], anti-CD49d [9F10], each at 10 μg/ml) for 30 min prior to assay (mean ± SEM, n = 3 per group; ^∗^p < 0.05, ^∗∗^p < 0.01). (C) The distribution of the indicated F-actin (green) and α-tubulin (red) was monitored by immunofluorescence and microscopy (after 4 days). The small box represents a high-resolution image. Scale bar = 100 μm. (D) Quantitative analysis of F-actin^+^ cells (mean ± SEM, n = 3 per group; ^∗∗^p < 0.01). (E) Quantitative analysis of cell width (n = 30) in RECs, MECs, and SECs (^∗∗^p < 0.01). (F) Imaging of transplanted cultured MSCs in vivo. A Luciferase image of cell migration in the recipient animals at 1 day postinjection of 1 × 10^5^ cultured cells (infected with Venus-ffLuc lentivirus) is shown. (G) Quantitation of luciferase activity in recipient animals. Luminescent intensity was significantly increased after injection of the cultured MSCs and MECs/SECs in lung regions (mean ± SEM, n = 3 per group; ^∗∗^p < 0.01).

VCAM-1 is known to bind to integrin α4β1 (VLA-4: CD49d/CD29), and this interaction is crucial for both rolling and adhesion of several cell types on the vascular endothelium prior to transendothelial trafficking (Papayannopoulou et al., 1998). A transwell migration assay revealed that RECs exhibited significantly greater migration ability than MECs or SECs; however, the motility of the RECs was abrogated in the presence of function-blocking antibodies directed against VCAM-1 or CD49d (Figures 6A and 6B). Microfilaments anchor to the cell membrane and are involved in cytoskeleton organization to produce effective cell motility (Gumbiner, 1996). F-actin is a major component of the microfilaments that are formed by polymerization of monomeric actin (G-actin). The formation of F-actin is balanced by an equilibrium reaction of G-actin incorporation and dissociation; typically, however, nonmotile cells accumulate large bundles of microfilaments known as stress fibers (Schratt et al., 2002). We therefore analyzed stress-fiber formation among the hMSC subgroups. Large tubular components (F-actin^+^) were detected in MECs and SECs, but not in RECs (Figure 6C). After cell culture with blocking antibody (anti-VCAM-1 or anti-CD49d), REC morphology was changed and stress-fiber components were increased (Figures 6C and 6D). Therefore, the enhanced migration capabilities of RECs may be associated with expression of VCAM-1 and CD49d. The formation of stress fibers is often accompanied by morphological changes of cells, typically characterized by enlargement of the cytoplasm (Wada et al., 2011). A comparison of cell widths (minor axis) clearly revealed that one REC cell was smaller than the others (Figure 6E). Notably, the cell width of RECs increased in response to VCAM-1 or CD49 blockade (Figure 6E).

A recent report suggested that artificial overexpression of LLP2A (ligand of CD49d) on hMSCs increases their migration to murine BM (Guan et al., 2012). Interestingly, VCAM-1 is also an equally effective ligand for CD49d. Following systemic infusion of RECs into murine recipients, there was no evidence of focal lung trapping, confirming enhanced motility. In contrast, MEC/SECs were largely sequestered in the pulmonary vasculature (Figures 6F and 6G). These observations led us to speculate that VCAM-1 expression may be essential to maintain the RECs’ morphology and thus their in vitro and in vivo motility.

### Further Purification of Functional MSCs

**Figure 7:**
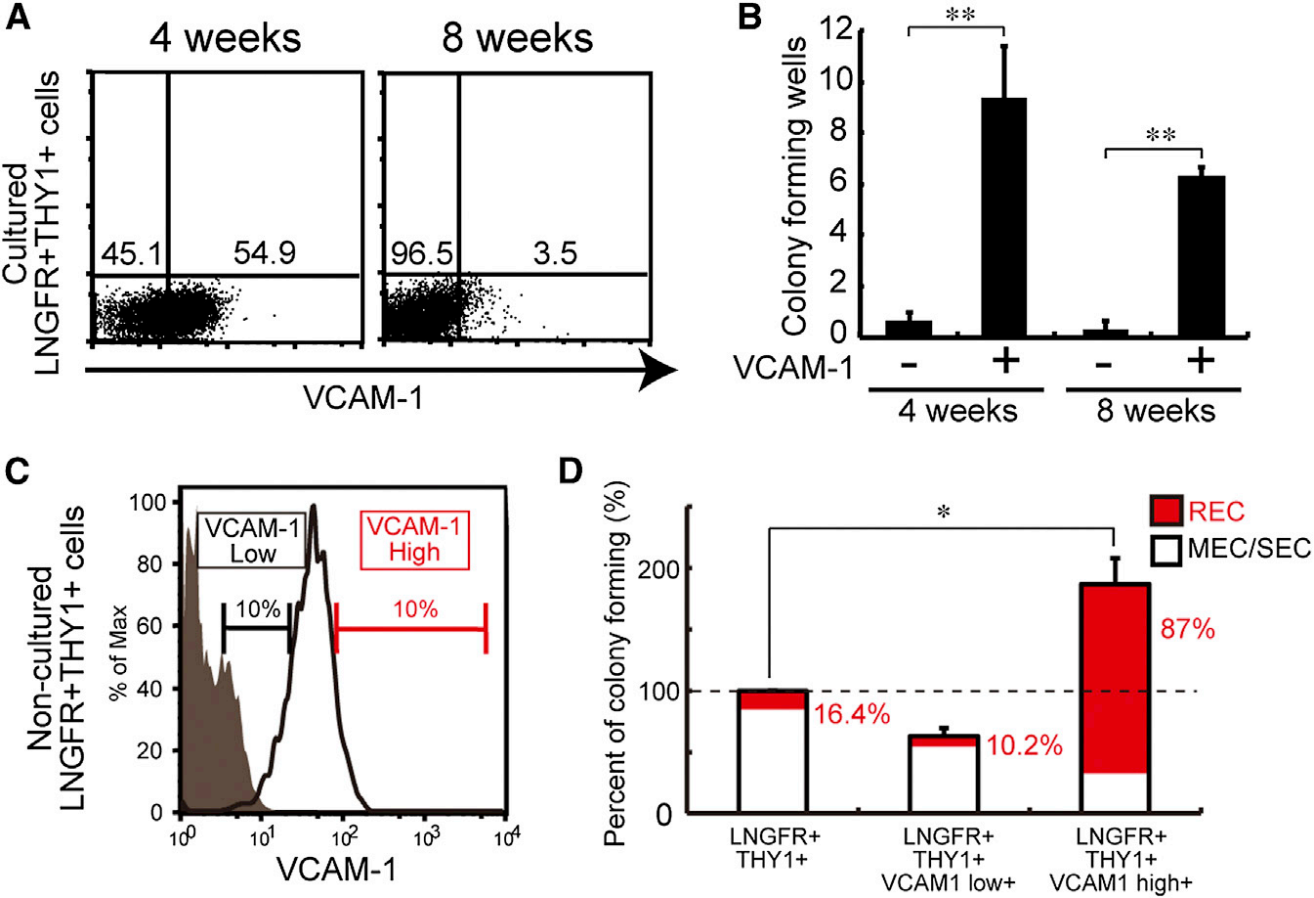
Isolation of Functional MSCs in Culture and Fresh BM Cells (A) Culture-dependent reduction in the frequency of VCAM-1^+^ cells in the LNGFR^+^THY-1^+^ cell population. (B) Colony-forming potential of cultured VCAM-1^+^ or VCAM-1^−^ cells derived from the LNGFR^+^THY-1^+^ cell population after 4 weeks and 8 weeks (mean ± SEM, n = 5; ^∗∗^p < 0.01). (C) Direct isolation of RECs from fresh BM cells. (D) Colony-forming potential of low-positive or high-positive VCAM-1 cells in the LNGFR^+^THY-1^+^ cell population (mean ± SEM, n = 3; ^∗^p < 0.05).

Using VCAM-1 as a specific marker, we attempted to isolate RECs from cultured MSCs or directly from fresh BM cells. Based on flow cytometry, freshly isolated LNGFR^+^THY-1^+^ cells uniformly expressed VCAM-1 (Figure 2G), but this gradually decreased with time during ex vivo expansion (Figure 7A). We then resorted VCAM-1^+^ and VCAM-1^−^ cells and compared the CFU-Fs of each cultured fraction. We found that VCAM-1^+^ cells had a far greater clonogenicity compared with VCAM-1^−^ cells (Figure 7B). We then examined the utility of VCAM-1 for isolating RECs directly from fresh WBM (Figure 7C). After single-cell sorting into 96-well plates, LNGFR^+^THY-1^+^VCAM-1^hi+^ (LTV) cells showed 2-fold higher CFU-Fs compared with LNGFR^+^THY-1^+^ (Figure 7D). Surprisingly, 87% of LTV CFU-Fs were RECs, whereas LNGFR^+^THY-1^+^- and LNGFR^+^THY-1^+^VCAM-1^lo+^-derived CFU-Fs contained far less RECs (16.4% and 10.2%, respectively; Figure 7D). Therefore, VCAM-1 can be used as a marker for enriching multipotent/proliferative cells (RECs) from both culture-expanded MSCs and fresh BM cells.

## Discussion

Despite the widespread clinical use of hMSCs, there are fundamental gaps in our knowledge of basic MSC biology. In contrast to HSCs, the molecular mechanisms that maintain MSCs in their undifferentiated state or determine their differentiation pathways are still poorly understood. Due to the limitations of the methods used to isolate MSCs, it has not been possible to obtain a sufficiently pure MSC population to address these questions. In the current study, we have demonstrated that LNGFR^+^THY-1^+^ cells in human BM constitute an extremely pure MSC population. The clonogenic potential of single-seeded LNGFR^+^THY-1^+^ MSCs, cultured in standard medium (Dulbecco’s modified Eagle’s medium [DMEM] + 20% fetal bovine serum [FBS]) exceeds that of other reported isolation methods (STRO-1^+^VCAM-1^+^ [Gronthos et al., 2003], MSCA-1^+^CD56^−^ [Battula et al., 2009], LNGFR^+^CD140b^+^ [Bühring et al., 2007], and LNGFR^+^CD146^+^ [Tormin et al., 2011]). Murine HSCs can be isolated based on CD34^−^, c-Kit^+^, Sca-1^+^, and Lineage^−^ expression, which yields one HSC per five cells (Matsuzaki et al., 2004). Our isolation method for hMSCs achieves a level of purity similar to that of mouse HSCs. Moreover, because LNGFR^+^THY-1^+^ cells contain the least number of hematopoietic cells, this combination enables the convenient isolation of MSCs based on the surface expression of only two antigens. This simple and easy isolation method should facilitate further studies and improve our basic understanding of the primary characteristics of MSCs.

Using highly enriched primary MSCs, one can perform clonal experiments easily and efficiently. Several groups have reported that MSCs are heterogeneous with respect to their morphological appearance (Kucia et al., 2007a, 2007b; Pittenger et al., 1999; Prockop et al., 2001). Our findings confirm that CFU-Fs derived from a single LNGFR^+^THY-1^+^ cell can be divided into three functionally distinct subpopulations termed RECs, MECs, and SECs. RECs exhibited robust multilineage differentiation and self-renewal potency. We therefore consider RECs to be the most primitive and undifferentiated population in CFU-Fs. In a previous report, upregulation of *p16Ink4a* and *p19Arf* by loss of the transcriptional repressor *Bmi-1* led to defective self-renewal of adult HSCs and neural stem cells, but was less critical for generating the differentiated progeny of these cell types (Park et al., 2003; Molofsky et al., 2003). In our results, MECs and SECs had increased expression of the two commonly known biomarkers for cellular senescence, SA-β-gal and *P16INK4a*. In contrast, *P14ARF*, which is generated by alternative splicing from the *INK4a* locus and *P21*, was not affected. We therefore speculate that the self-renewal capacity of cultured MSCs is mediated by an unknown mechanism rather than by *BMI-1* regulation.

VCAM-1 encodes a leukocyte adhesion molecule whose expression is restricted to endothelial cells and subpopulations of BM cells (Osborn et al., 1989). VCAM-1 binds to the integrin α4β1 and integrin α4β7 on circulating monocytes, granulocytes, and lymphocytes (Elices et al., 1990; Osborn et al., 1989). Increased expression of VCAM-1 has been reported in various cancer cell types, including breast, gastric, renal carcinoma, and melanomas (Ding et al., 2003; Minn et al., 2005). It has been suggested that the VCAM-1-CD49d interaction promotes cancer cell survival and migration (Klemke et al., 2007). In this study, we demonstrated that RECs have higher expression of VCAM-1 compared with MECs or SECs, and blockade of the VCAM-1-CD49d interaction significantly diminished transwell migration and increased stress-fiber formation. The functional difference between the groups relates to the organization of the F-actin cytoskeleton, since the rigid cytoskeletal structure inhibits their ability to transmigrate across the vascular endothelium (Morales-Ruiz et al., 2000). Therefore, we speculate that RECs interact with each other through VCAM-1-CD49d, thereby maintaining their primitive state, including self-renewal, multilineage potential, and migratory capacity.

In this study we have described methods for the selective isolation of primitive hMSCs that may prove advantageous both experimentally and therapeutically. The identification of the combination marker LTV makes it possible to prospectively isolate functional hMSCs directly from BM. Using these cells, we may be able to tease out the complex molecular mechanisms that govern the undifferentiated state of hMSCs. Additionally, these markers may allow visualization of hMSCs in vivo, thereby helping to uncover their physiological and pathophysiological roles. The use of VCAM-1 as an REC-selective marker facilitates the isolation of enriched MSCs from a heterogeneous population of cultured MSCs that represents mixed CFU-Fs. Since we demonstrated that the coexistence of SECs alters the self-renewal and differentiation potency of RECs in culture, the isolation of VCAM-1^+^ cells from cultured hMSCs may prove useful therapeutically. In conclusion, our data should help to unravel the fundamental cellular and molecular biology of MSCs. This information is critical if MSCs are to be used effectively and safely in regenerative therapy.

## Experimental Procedures

### Tissue Collection

Heads of femurs were collected from patients (22–80 years old) who had undergone hip replacement arthroplasty at Keio University Hospital, Tokyo. In total, 23 biological samples were obtained and used for the experiments described herein. Placenta, fat, PB, and UCB samples were also collected and stored in accordance with Keio University protocols and guidelines. Approval for the study was obtained from the Research Ethics Committee of the Keio University School of Medicine (No. 18-26), and patient recruitment was undertaken with written informed consent.

### Cell Preparation

The heads of femurs were dissected and crushed with a pestle, after which the crushed bones were washed gently once in PBS to collect BM cells. The bone fragments were incubated for 2 hr at 37°C in DMEM (Invitrogen) in the presence of 0.2% collagenase (cell dissociation grade; Wako Chemicals) and 25 μg/ml deoxyribonuclease I (Sigma-Aldrich) to yield a suspension of CR cells. The CR and BM cells were then used in the initial experiments to define prospective markers for the identification of MSCs. Placenta and fat-tissue experiments were also used for BM isolation protocols as described in the Supplemental Experimental Procedures.

### Antibody Staining and Flow Cytometry

Cells were suspended in ice-cold Hank’s balanced salt solution at 1–5 × 10^7^ cells/ml and then stained for 30 min on ice with a monoclonal antibody as described in the Supplemental Experimental Procedures. The antibodies used were LNGFR-PE (Miltenyi Biotec), THY-1-FITC, and VCAM-1-APC (BD PharMingen, Biolegend). Flow-cytometric analysis and sorting were performed on a triple-laser MoFlo (Beckman Coulter), FACSVantage SE, or FACSCalibur flow cytometer (Becton Dickinson), and the data were analyzed using Flowjo software (Tree Star). Analysis of cell populations expressing varying combinations of LNGFR and THY-1 on their surface from the different tissues routinely demonstrated 99% purity by flow cytometry.

### Limiting Dilution Assays

The sorted cells were seeded at different concentrations into 96-well plates. After 14 days, wells with no colonies were included in the count for each population. The concentration of plated cells that resulted in 37% of the wells being negative for colony growth was taken as the cell concentration that yielded one CFU-F/well (Bacon and Sytkowski, 1987). Populations of sorted cells that were >99% pure were routinely prepared.

### Single-Cell Sorting and Secondary CFU-F Assays

Flow-cytometric analyses and the collection of single cells for CFU-F assays were carried out using a FACSVantage SE cytometer. The cells were sorted directly into separate wells of a 96-well plate containing 200 μl of medium using a CloneCyt automated cell deposition unit. Calibration of the clone sorting using fluorescent beads showed that <1% of the wells received more or less than one bead. Secondary CFU-F assays were performed by flow cytometry, seeding single propidium iodide (PI)-negative cells (living cells) from a starting population of cultured cells.

### DNA Preparation and Quality Assessments

Genomic DNA was isolated using a QIAGEN DNeasy Blood & Tissue Kit and Agilent’s recommended procedure from 5 × 10^6^ cells (RECs, MECs, and SECs at 6 weeks) as described in the Supplemental Experimental Procedures.

### Oligonucleotide aCGH Analysis

aCGH experiments were performed according to the manufacturer’s protocol (Agilent oligonucleotide array-based CGH for genomic DNA analysis, version 6.0, direct method) as described in the Supplemental Experimental Procedures. All aCGH data are available for viewing at the Gene Expression Omnibus (GEO) under accession number GSE34484.

### Index Sorting Assay

Index sorting was carried out according to the instrument user’s guide (FACSVantage SE option and assembly; Becton Dickinson Biosciences). Index sorting facilitates the linkage of marker expression information from an individual cell with its flow-cytometric profile.

### Migration Assays

Migration assays were performed using polyethylene terephthalate filters with 8 μm pores (BD Biocoat) separating the upper and lower chambers as described in the Supplemental Experimental Procedures.

### Imaging of Transplanted Cultured MSCs In Vivo

RECs, MECs/SECs, and cultured MSCs were expanded and then infected with Venus-ffLuc lentivirus (the Venus fluorescent and luminescent fusion protein; Hara-Miyauchi et al., 2012). Venus^+^-infected cells (1 × 10^5^ cells) were then sorted and transplanted intravenously. One day postinjection, the treated mice were anesthetized and given D-luciferin (150 mg/kg body weight) i.p. To quantify the measured light, regions of interest (ROIs) were defined as lungs, and all values were examined from an equal ROI by Xenogen-IVIS 100-cooled CCD optical macroscopic imaging system (SC BioScience) as described in the Supplemental Experimental Procedures.

### Statistical Analysis

Quantitative data are presented as the mean ± SEM from representative experiments (n ≥ 3). For the statistical analysis, the data were evaluated with a Student’s t test; p values < 0.05 were considered significant.

## Accession Numbers

The Gene Expression Omnibus accession number for CGH array reported in this paper is GSE34484.

## Supplemental Information

Document S1. Supplemental Experimental Procedures, Figures S1–S5, and Tables S1 and S2

## References

[bib1] Aslan H., Zilberman Y., Kandel L., Liebergall M., Oskouian R.J., Gazit D., Gazit Z. (2006). Osteogenic differentiation of noncultured immunoisolated bone marrow-derived CD105+ cells. Stem Cells.

[bib2] Bacon E.R., Sytkowski A.J. (1987). Identification and characterization of a differentiation-specific antigen on normal and malignant murine erythroid cells. Blood.

[bib3] Battula V.L., Bareiss P.M., Treml S., Conrad S., Albert I., Hojak S., Abele H., Schewe B., Just L., Skutella T. (2007). Human placenta and bone marrow derived MSC cultured in serum-free, b-FGF-containing medium express cell surface frizzled-9 and SSEA-4 and give rise to multilineage differentiation. Differentiation.

[bib4] Battula V.L., Treml S., Bareiss P.M., Gieseke F., Roelofs H., de Zwart P., Müller I., Schewe B., Skutella T., Fibbe W.E. (2009). Isolation of functionally distinct mesenchymal stem cell subsets using antibodies against CD56, CD271, and mesenchymal stem cell antigen-1. Haematologica.

[bib5] Ben-David U., Mayshar Y., Benvenisty N. (2011). Large-scale analysis reveals acquisition of lineage-specific chromosomal aberrations in human adult stem cells. Cell Stem Cell.

[bib6] Bianco P., Cao X., Frenette P.S., Mao J.J., Robey P.G., Simmons P.J., Wang C.Y. (2013). The meaning, the sense and the significance: translating the science of mesenchymal stem cells into medicine. Nat. Med..

[bib7] Boiret N., Rapatel C., Veyrat-Masson R., Guillouard L., Guérin J.J., Pigeon P., Descamps S., Boisgard S., Berger M.G. (2005). Characterization of nonexpanded mesenchymal progenitor cells from normal adult human bone marrow. Exp. Hematol..

[bib8] Bühring H.J., Battula V.L., Treml S., Schewe B., Kanz L., Vogel W. (2007). Novel markers for the prospective isolation of human MSC. Ann. N Y Acad. Sci..

[bib9] Ding Y.B., Chen G.Y., Xia J.G., Zang X.W., Yang H.Y., Yang L. (2003). Association of VCAM-1 overexpression with oncogenesis, tumor angiogenesis and metastasis of gastric carcinoma. World J. Gastroenterol..

[bib10] Drost A.C., Weng S., Feil G., Schäfer J., Baumann S., Kanz L., Sievert K.D., Stenzl A., Möhle R. (2009). In vitro myogenic differentiation of human bone marrow-derived mesenchymal stem cells as a potential treatment for urethral sphincter muscle repair. Ann. N Y Acad. Sci..

[bib11] Elices M.J., Osborn L., Takada Y., Crouse C., Luhowskyj S., Hemler M.E., Lobb R.R. (1990). VCAM-1 on activated endothelium interacts with the leukocyte integrin VLA-4 at a site distinct from the VLA-4/fibronectin binding site. Cell.

[bib12] Erices A., Conget P., Minguell J.J. (2000). Mesenchymal progenitor cells in human umbilical cord blood. Br. J. Haematol..

[bib13] Friedenstein A.J., Deriglasova U.F., Kulagina N.N., Panasuk A.F., Rudakowa S.F., Luriá E.A., Ruadkow I.A. (1974). Precursors for fibroblasts in different populations of hematopoietic cells as detected by the in vitro colony assay method. Exp. Hematol..

[bib14] Galderisi U., Helmbold H., Squillaro T., Alessio N., Komm N., Khadang B., Cipollaro M., Bohn W., Giordano A. (2009). In vitro senescence of rat mesenchymal stem cells is accompanied by downregulation of stemness-related and DNA damage repair genes. Stem Cells Dev..

[bib15] Galvin K.A., Jones D.G. (2002). Adult human neural stem cells for cell-replacement therapies in the central nervous system. Med. J. Aust..

[bib16] Gronthos S., Zannettino A.C., Hay S.J., Shi S., Graves S.E., Kortesidis A., Simmons P.J. (2003). Molecular and cellular characterisation of highly purified stromal stem cells derived from human bone marrow. J. Cell Sci..

[bib17] Guan M., Yao W., Liu R., Lam K.S., Nolta J., Jia J., Panganiban B., Meng L., Zhou P., Shahnazari M. (2012). Directing mesenchymal stem cells to bone to augment bone formation and increase bone mass. Nat. Med..

[bib18] Guillot P.V., Abass O., Bassett J.H., Shefelbine S.J., Bou-Gharios G., Chan J., Kurata H., Williams G.R., Polak J., Fisk N.M. (2008). Intrauterine transplantation of human fetal mesenchymal stem cells from first-trimester blood repairs bone and reduces fractures in osteogenesis imperfecta mice. Blood.

[bib19] Gumbiner B.M. (1996). Cell adhesion: the molecular basis of tissue architecture and morphogenesis. Cell.

[bib20] Hara-Miyauchi C., Tsuji O., Hanyu A., Okada S., Yasuda A., Fukano T., Akazawa C., Nakamura M., Imamura T., Matsuzaki Y. (2012). Bioluminescent system for dynamic imaging of cell and animal behavior. Biochem. Biophys. Res. Commun..

[bib21] Houlihan D.D., Mabuchi Y., Morikawa S., Niibe K., Araki D., Suzuki S., Okano H., Matsuzaki Y. (2012). Isolation of mouse mesenchymal stem cells on the basis of expression of Sca-1 and PDGFR-α. Nat. Protoc..

[bib23] Jung Y., Bauer G., Nolta J.A. (2012). Concise review: Induced pluripotent stem cell-derived mesenchymal stem cells: progress toward safe clinical products. Stem Cells.

[bib24] Kim J., Kang J.W., Park J.H., Choi Y., Choi K.S., Park K.D., Baek D.H., Seong S.K., Min H.K., Kim H.S. (2009). Biological characterization of long-term cultured human mesenchymal stem cells. Arch. Pharm. Res..

[bib25] Klemke M., Weschenfelder T., Konstandin M.H., Samstag Y. (2007). High affinity interaction of integrin alpha4beta1 (VLA-4) and vascular cell adhesion molecule 1 (VCAM-1) enhances migration of human melanoma cells across activated endothelial cell layers. J. Cell. Physiol..

[bib26] Kucia M., Halasa M., Wysoczynski M., Baskiewicz-Masiuk M., Moldenhawer S., Zuba-Surma E., Czajka R., Wojakowski W., Machalinski B., Ratajczak M.Z. (2007). Morphological and molecular characterization of novel population of CXCR4+ SSEA-4+ Oct-4+ very small embryonic-like cells purified from human cord blood: preliminary report. Leukemia.

[bib27] Kucia M., Wu W., Ratajczak M.Z. (2007). Bone marrow-derived very small embryonic-like stem cells: their developmental origin and biological significance. Dev. Dyn..

[bib28] Lee O.K., Kuo T.K., Chen W.M., Lee K.D., Hsieh S.L., Chen T.H. (2004). Isolation of multipotent mesenchymal stem cells from umbilical cord blood. Blood.

[bib29] Matsuzaki Y., Kinjo K., Mulligan R.C., Okano H. (2004). Unexpectedly efficient homing capacity of purified murine hematopoietic stem cells. Immunity.

[bib30] Minn A.J., Gupta G.P., Siegel P.M., Bos P.D., Shu W., Giri D.D., Viale A., Olshen A.B., Gerald W.L., Massagué J. (2005). Genes that mediate breast cancer metastasis to lung. Nature.

[bib22] Molofsky A.V., Pardal R., Iwashita T., Park I.K., Clarke M.F., Morrison S.J. (2003). Bmi-1 dependence distinguishes neural stem cell self-renewal from progenitor proliferation. Nature.

[bib31] Morales-Ruiz M., Fulton D., Sowa G., Languino L.R., Fujio Y., Walsh K., Sessa W.C. (2000). Vascular endothelial growth factor-stimulated actin reorganization and migration of endothelial cells is regulated via the serine/threonine kinase Akt. Circ. Res..

[bib32] Morikawa S., Mabuchi Y., Kubota Y., Nagai Y., Niibe K., Hiratsu E., Suzuki S., Miyauchi-Hara C., Nagoshi N., Sunabori T. (2009). Prospective identification, isolation, and systemic transplantation of multipotent mesenchymal stem cells in murine bone marrow. J. Exp. Med..

[bib33] Nagase T., Ueno M., Matsumura M., Muguruma K., Ohgushi M., Kondo N., Kanematsu D., Kanemura Y., Sasai Y. (2009). Pericellular matrix of decidua-derived mesenchymal cells: a potent human-derived substrate for the maintenance culture of human ES cells. Dev. Dyn..

[bib34] Osborn L., Hession C., Tizard R., Vassallo C., Luhowskyj S., Chi-Rosso G., Lobb R. (1989). Direct expression cloning of vascular cell adhesion molecule 1, a cytokine-induced endothelial protein that binds to lymphocytes. Cell.

[bib35] Papayannopoulou T., Priestley G.V., Nakamoto B. (1998). Anti-VLA4/VCAM-1-induced mobilization requires cooperative signaling through the kit/mkit ligand pathway. Blood.

[bib51] Park I.K., Qian D., Kiel M., Becker M.W., Pihalja M., Weissman I.L., Morrison S.J., Clarke M.F. (2003). Bmi-1 is required for maintenance of adult self-renewing haematopoietic stem cells. Nature.

[bib36] Pittenger M.F., Mackay A.M., Beck S.C., Jaiswal R.K., Douglas R., Mosca J.D., Moorman M.A., Simonetti D.W., Craig S., Marshak D.R. (1999). Multilineage potential of adult human mesenchymal stem cells. Science.

[bib37] Prockop D.J., Sekiya I., Colter D.C. (2001). Isolation and characterization of rapidly self-renewing stem cells from cultures of human marrow stromal cells. Cytotherapy.

[bib38] Quirici N., Soligo D., Bossolasco P., Servida F., Lumini C., Deliliers G.L. (2002). Isolation of bone marrow mesenchymal stem cells by anti-nerve growth factor receptor antibodies. Exp. Hematol..

[bib39] Rombouts W.J., Ploemacher R.E. (2003). Primary murine MSC show highly efficient homing to the bone marrow but lose homing ability following culture. Leukemia.

[bib40] Sacchetti B., Funari A., Michienzi S., Di Cesare S., Piersanti S., Saggio I., Tagliafico E., Ferrari S., Robey P.G., Riminucci M., Bianco P. (2007). Self-renewing osteoprogenitors in bone marrow sinusoids can organize a hematopoietic microenvironment. Cell.

[bib41] Schratt G., Philippar U., Berger J., Schwarz H., Heidenreich O., Nordheim A. (2002). Serum response factor is crucial for actin cytoskeletal organization and focal adhesion assembly in embryonic stem cells. J. Cell Biol..

[bib42] Simmons P.J., Torok-Storb B. (1991). Identification of stromal cell precursors in human bone marrow by a novel monoclonal antibody, STRO-1. Blood.

[bib43] Spits C., Mateizel I., Geens M., Mertzanidou A., Staessen C., Vandeskelde Y., Van der Elst J., Liebaers I., Sermon K. (2008). Recurrent chromosomal abnormalities in human embryonic stem cells. Nat. Biotechnol..

[bib44] Stappenbeck T.S., Miyoshi H. (2009). The role of stromal stem cells in tissue regeneration and wound repair. Science.

[bib45] Tao X.R., Li W.L., Su J., Jin C.X., Wang X.M., Li J.X., Hu J.K., Xiang Z.H., Lau J.T., Hu Y.P. (2009). Clonal mesenchymal stem cells derived from human bone marrow can differentiate into hepatocyte-like cells in injured livers of SCID mice. J. Cell. Biochem..

[bib46] Tormin A., Li O., Brune J.C., Walsh S., Schütz B., Ehinger M., Ditzel N., Kassem M., Scheding S. (2011). CD146 expression on primary nonhematopoietic bone marrow stem cells is correlated with in situ localization. Blood.

[bib47] Wada K., Itoga K., Okano T., Yonemura S., Sasaki H. (2011). Hippo pathway regulation by cell morphology and stress fibers. Development.

[bib48] Yen B.L., Huang H.I., Chien C.C., Jui H.Y., Ko B.S., Yao M., Shun C.T., Yen M.L., Lee M.C., Chen Y.C. (2005). Isolation of multipotent cells from human term placenta. Stem Cells.

[bib49] Zimmerlin L., Donnenberg V.S., Pfeifer M.E., Meyer E.M., Peault B., Rubin J.P., Donnenberg A.D. (2010). Stromal vascular progenitors in adult human adipose tissue. Cytometry A.

[bib50] Zvaifler N.J., Marinova-Mutafchieva L., Adams G., Edwards C.J., Moss J., Burger J.A., Maini R.N. (2000). Mesenchymal precursor cells in the blood of normal individuals. Arthritis Res..

