# Supplementary material for: LNGFR+THY-1+VCAM-1hi+ Cells Reveal Functionally Distinct Subpopulations in Mesenchymal Stem Cells
Source: Stem Cell Reports. 2013 Jul 11;1(2):152–65. doi: 10.1016/j.stemcr.2013.06.001 (PMC3757748; doi:10.1016/j.stemcr.2013.06.001)
Supplement: Document S1. Supplemental Experimental Procedures, Figures S1–S5, and Tables S1 and S2 [file mmc1.pdf]

Stem Cell Reports, volume *1*

Supplemental Information

LNGFR^{+}THY-1^{+}VCAM-1^{hi+} Cells Reveal Functionally Distinct Subpopulations in Mesenchymal Stem Cells

Yo Mabuchi, Satoru Morikawa, Seiko Harada, Kunimichi Niibe, Sadafumi Suzuki, Francois Renault-Mihara, Diarmaid D. Houlihan, Chihiro Akazawa, Hideyuki Okano, and Yumi Matsuzaki

INVENTORY OF SUPPLEMENTAL INFORMATION

SUPPLEMENTAL FIGURES AND LEGENDS

・ Figure S1: Hematopoietic contamination of selected cell populations, Related to Figure 1.

・ Figure S2: LNGFR^{+}THY-1^{+} cells from placenta, adipose tissue and peripheral blood, Related to Figure 2.

・ Figure S3: Homing and multi-potential capacity of LNGFR^{+}THY-1^{+} cells *in vivo*, Related to Figure 2.

・ Figure S4: REC growth kinetics are influenced by other subpopulations, Related to Figure 4.

・ Figure S5: Expression of surface markers on RECs, MECs and SECs, Related to Figure 5.

・Table S1: Differentiation potential of RECs, MECs and SECs, Related to Figure 3.

・Table S2: Genomic abnormalities in RECs, MECs and SECs assessed using array-based comparative genomic hybridization, Related to Figure 4.

SUPPLEMENTAL EXPERIMENTAL PROCEDURES

SUPPLEMENTAL REFERENCES

SUPPLEMENTAL INFORMATION

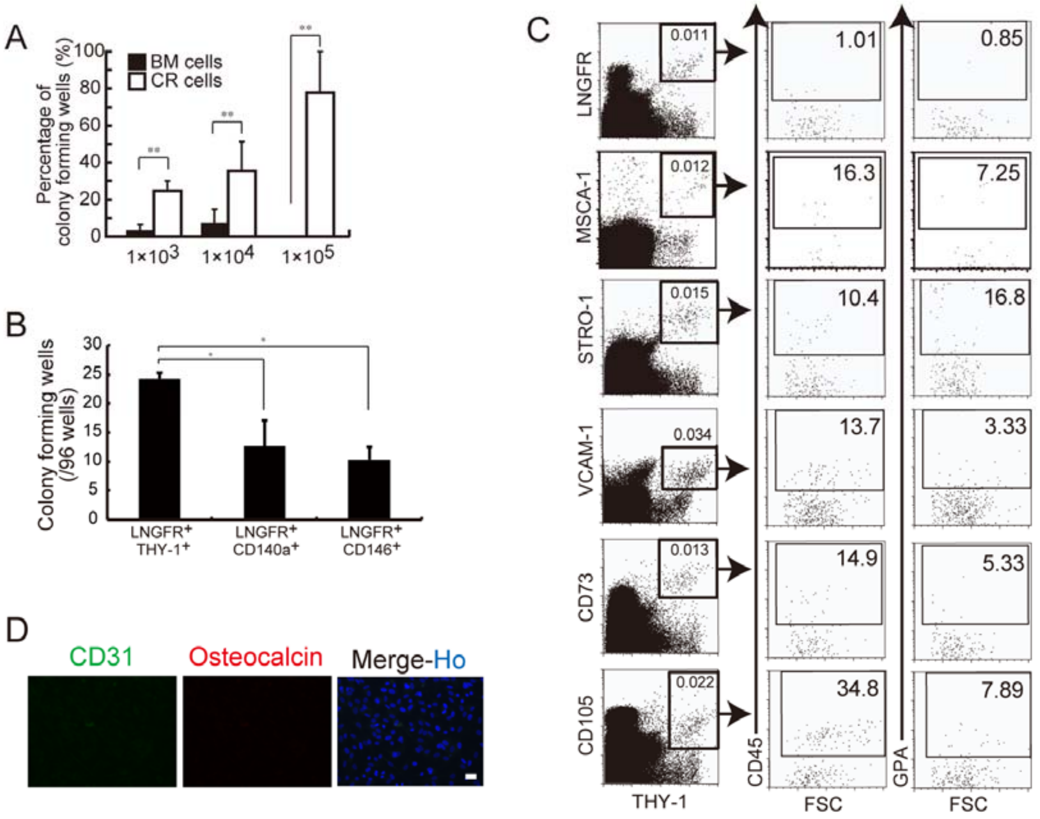

Figure S1: Hematopoietic contamination of selected cell populations, Related to Figure 1.

(A) Colony-forming assay of BM and CR cells after 14 days in culture. BM and CR cells were seeded at the indicated cell number in 96-well culture plates (mean ± s.e.m., n = 3) (**P < 0.01).

(B) Single cell sorting assay to directly compare CFU-F potential in LNGFR^{+}THY-1^{+}, LNGFR^{+}CD140a^{+} and LNGFR^{+}CD146^{+} sub-populations (mean ± s.e.m., n = 3) (*P < 0.05).

(C) Identification of contaminating hematopoietic cells (CD45^{+} or Glycophorin A: GPA^{+}) using flow cytometry in selected populations isolated from WBM based on the positive expression of THY-1, LNGFR, MSCA-1, STRO-1, VCAM-1, CD73 and CD105.

(D) The LNGFR^{+}THY-1^{+} population is not contaminated by endothelial cells (CD31 staining negative) and osteogenic cells (Osteocalcin staining negative). Scale bar = 100 μm.

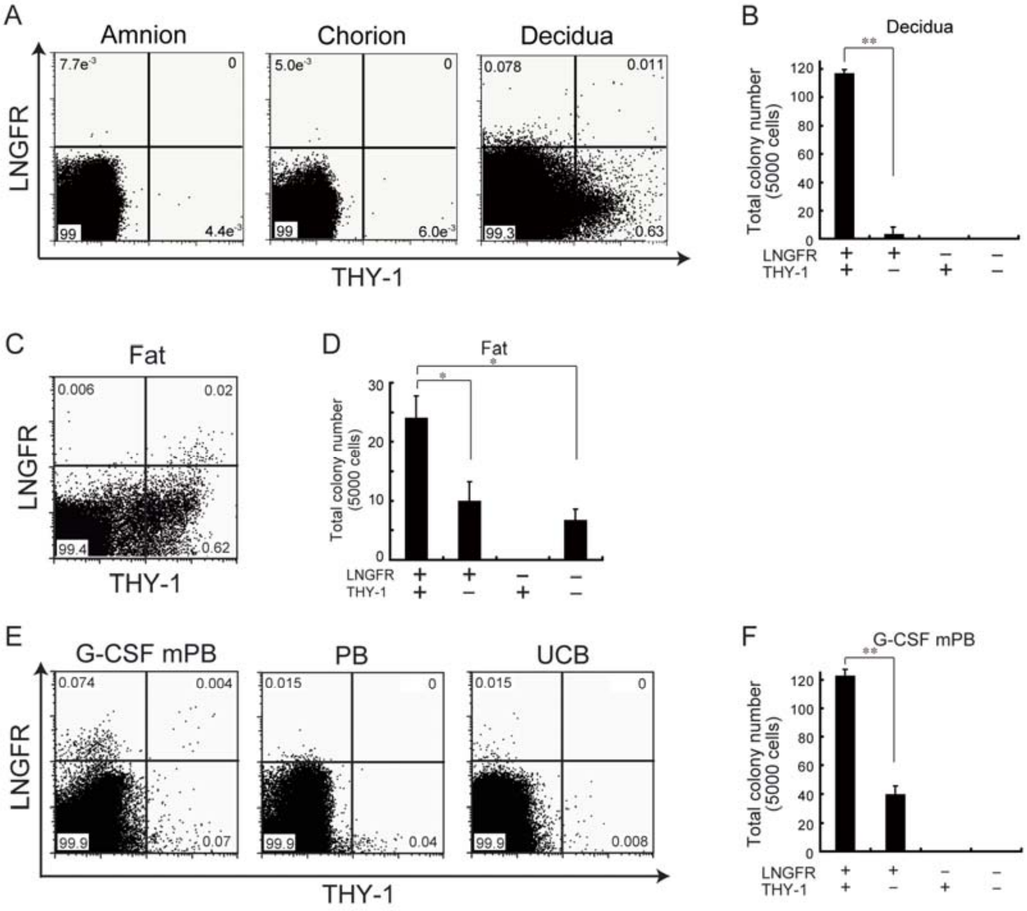

Figure S2: LNGFR$^+$THY-1$^+$ cells from placenta, adipose tissue and peripheral blood, Related to Figure 2.

(A) Flow cytometric analysis of placental tissue (amnion, chorion and decidua) stained for LNGFR and THY-1. (B) Numbers of CFU-Fs 14 days after plating 5000 decidua-derived LNGFR$^+$/THY-1$^+$, +/-, -/+ and -/- cells (mean ± s.e.m., n = 3) (**P < 0.01). (C) Flow cytometric profiles of adipose tissue stained for LNGFR and THY-1. (D) Numbers of CFU-Fs 14 days after plating 5000 adipose-derived LNGFR$^+$THY-1$^+$ cells (mean ± s.e.m., n = 3) (*P < 0.05). (E) Identification of LNGFR$^+$THY-1$^+$ cells from granulocyte-colony stimulating factor-mobilized peripheral blood (G-CSF mPB), peripheral blood (PB) and umbilical cord blood (UCB). (F) Numbers of CFU-Fs 14 days after plating 5000 G-CSF mPB -derived LNGFR$^+$THY-1$^+$ cells (mean ± s.e.m., n = 3) (**P < 0.01).

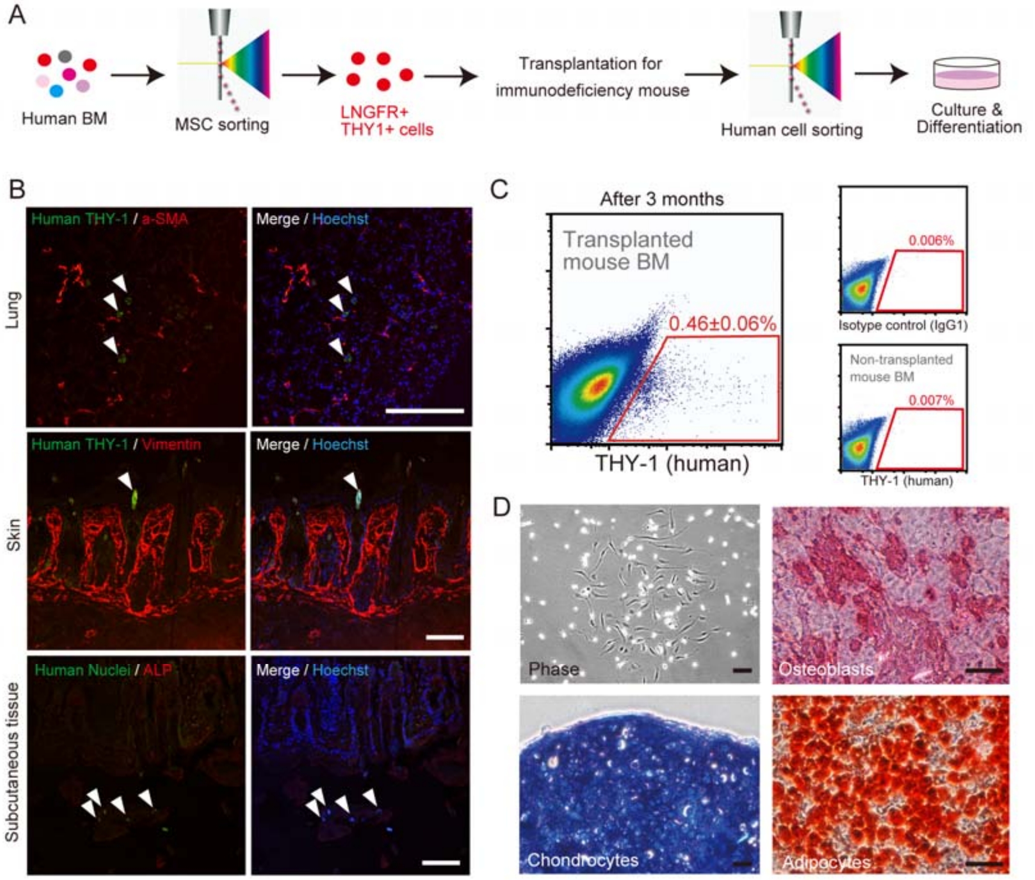

Figure S3: Homing and multi-potential capacity of LNGFR$^+$THY-1$^+$ cells *in vivo*, Related to Figure 2.

(A) The LNGFR$^+$THY-1$^+$ cells were isolated from human BM (BM-MNCs POIETICS) and xeno-transplanted into immunodeficient (NOG) mice. After three months, NOG mouse BM was analyzed by immunohistochemistry and flow cytometer. (B) Immunocytochemistry of transplanted mouse tissue with anti-human antibody. The human antibody is highly selective in distinguishing human from mouse species (Anti-THY-1 and anti-Human Nuclear Antigen). Hoechst stain for nuclei. Scale bar = 200 μm. (C) Flow cytometric analysis of BM from NOG mice by anti-human THY-1. The left plot demonstrates positive expression of human THY-1 indicating engraftment of the human cells into the murine BM (mean ± s.e.m., n = 4). The right plots show isotype control (IgG1) (right top) and human antibody staining in non-transplanted mouse BM (right bottom). (D) The isolated human THY-1$^+$ fraction formed CFU-F in culture (Phase). Human THY-1$^+$ cells differentiated into mesenchymal lineages (osteocytes, chondrocytes and adipocytes). Data are representative of three independent experiments. Scale bar = 100 μm.

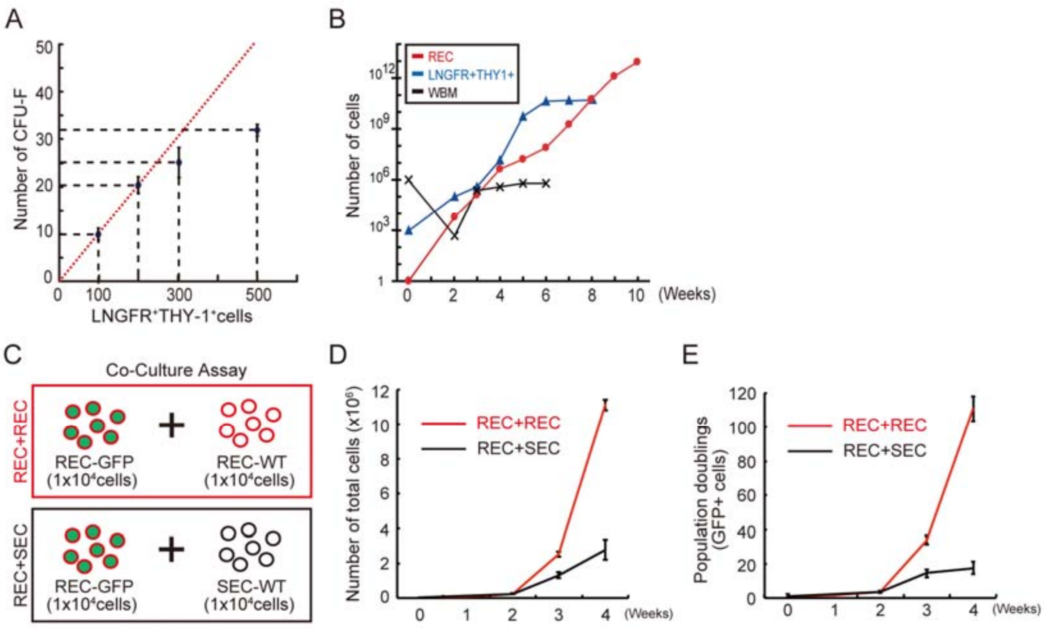

Figure S4: REC growth kinetics are influenced by other subpopulations, Related to Figure 4.

(A) Dose-response curve illustrating the change in the number of CFU-Fs generated with increasing numbers of seeded LNGFR$^+$THY-1$^+$ cells. (B) Growth curves for RECs, LNGFR$^+$THY-1$^+$ cells and WBM (n = 3 per group). (C-E) Co-culture Assay. (C) GFP-transduced RECs via a lentiviral vector (REC-GFP) were co-cultured with REC-WT (non-GFP) or SEC-WT (non-GFP) *in vitro* (1 x 10^4 cells at 4 weeks). (D) Total cell numbers of REC-GFP with REC-WT (REC+REC: red line) and REC-GFP with SEC-WT (REC+SEC: black line) are shown (mean ± s.e.m., n = 3). (E) Population doublings (PD) for GFP-positive REC cells in the presence and absence of SEC cells are also shown (mean ± s.e.m., n = 3).

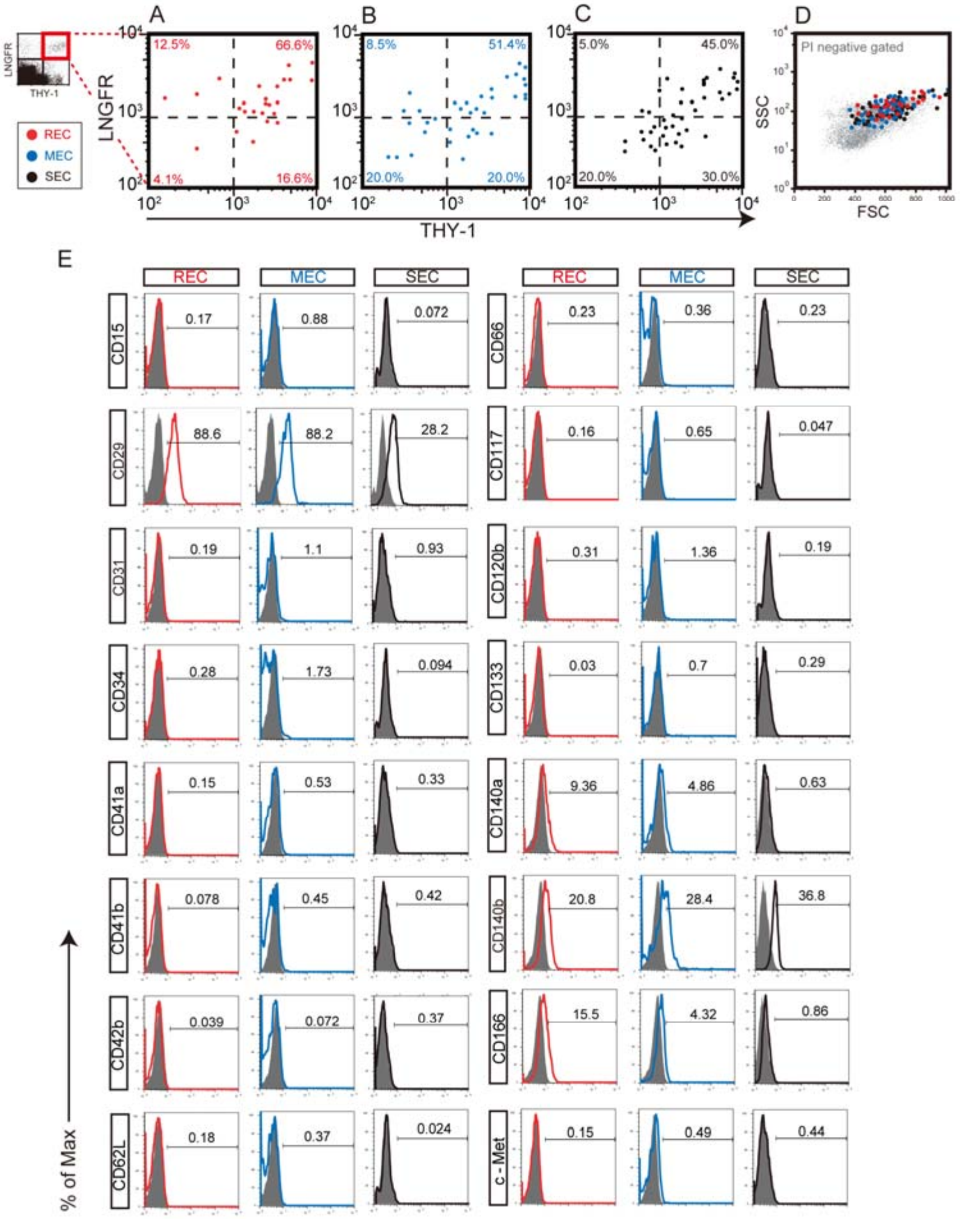

Figure S5: Expression of surface markers on RECs, MECs and SECs, Related to Figure 5.

(A-D) Index cell sorting demonstrating the distribution of cells in the LNGFR$^+$THY-1$^+$ fraction containing RECs (A), MECs (B), SECs (C) and Side scatter (SSC) vs. Forward scatter (FSC) profile (D). (E) Cell surface expression on RECs, MECs and SECs (six weeks in culture). Shown is the percentage of cells that express the antigen (line) vs. a matched isotype control (gray).

		Clone	ACO	AC	AO	CO	A	C	O	None
**REC**	%[a)]	100	80	9	11	0	0	0	0	0
	n[b)]	46	37	4	5	0	0	0	0	0
**MEC**	%[a)]	100	63	10	6	0	6	2	0	13
	n[b)]	48	30	5	3	0	3	1	0	6
**SEC**	%[a)]	100	25	6	31	0	0	6	18	14
	n[b)]	51	13	3	16	0	0	3	9	7

Table S1: Differentiation potential of RECs, MECs and SECs, Related to Figure 3. Single colonies (46 RECs, 48 MECs and 51 SECs) were expanded for one month, and further re-seeded and cultured under appropriate conditions for adipogenic, osteogenic and chondrogenic differentiation. The differentiation potential of each clone (n = 145) was examined individually. Each clone was then designated with the letter ACO (differentiation for adipocytes, chondrocytes and osteoblasts), AC (adipocytes, chondrocytes), AO (adipocytes, osteoblasts), CO (chondrocytes, osteoblasts), A (adipocytes), C (chondrocytes), O (osteoblasts) and None (not differentiation).

a) Ratio of corresponding clones for differentiation potential. b) Number of clones.

Clone	Number of aberrations [a]	Overlap CNVs [b]	Non-overlap CNVs [c]
REC-1	8	8	0
REC-2	12	12	0
REC-3	15	15	0
MEC-1	29	25	4
MEC-2	36	29	7
MEC-3	29	25	4
SEC-1	31	26	5
SEC-2	35	29	6
SEC-3	26	21	5

Table S2: Genomic abnormalities in RECs, MECs and SECs assessed using array-based comparative genomic hybridization, Related to Figure 4.

Numbers of copy number variations (CNVs) in RECs, MECs and SECs after six weeks in culture. a) Number of aberrations was counted by oligonucleotide array CGH analysis. b) Overlap CNVs that overlap with the Database of Genomic Variants (CNV_20090312) were counted. c) Non-overlap CNVs (Number of aberrations – Overlap CNVs) are formed *de novo* and generate genomic errors. The aCGH data are available for viewing at the Gene Expression Omnibus (GEO) under accession number GSE34484 (all aCGH data).

SUPPLEMENTAL EXPERIMENTAL PROCEDURES

Cell preparation

The heads of femurs were dissected and crushed with a pestle, after which the crushed bones were washed gently once in phosphate buffered saline (PBS) to collect BM cells. The bone fragments were incubated for 2 hours at 37°C in Dulbecco's Modified Eagle Medium (DMEM: Invitrogen) in the presence of 0.2% collagenase (Wako Chemicals, Cell dissociation grade) and 25 μg/ml deoxyribonuclease □ (Sigma-Aldrich), to yield a suspension of collagenase-released cells. The suspension was filtered through a cell strainer (Falcon 2350, 70 μm) to remove debris and bone fragments, after which the cells were pelleted by centrifugation at 280 *g* for 5 min at room temperature. Next, the cells were resuspended for 5 to 10 seconds in 1 ml of water to lyse the red blood cells. After adding 1 ml of 2 x PBS containing 4% fetal bovine serum (FBS) to quench the reaction, the remaining cells were resuspended in calcium- and magnesium-free Hank's Balanced Salt Solution (HBSS) (Gibco) supplemented with 2% FBS, 10 mM HEPES and 1% penicillin/streptomycin (P/S), and the suspension was filtered through a cell strainer to remove debris (CR cells). The CR and BM cells were then used in the initial experiments to define prospective markers for the identification of MSCs. Further experiments were carried out using BM-MNCs or BONE MARROW MNC (POIETICS™). Placenta and fat tissue experiments were also used BM isolation protocols.

Colony-forming assay

Different numbers of BM and CR cells were seeded into 96-well plates. After 14 days, each well was fixed in methanol and stained with Giemsa. Wells with colonies were included in the count for each cell type. CFU-F assays were performed by sorting cells into a 100 mm dish and culturing them for 14 days in standard culture medium, which consisted of DMEM supplemented with 20% FBS (Hyclone), 20 ng/ml bFGF (Peprotech) and 1% P/S, and which was changed twice weekly. Thereafter, colonies containing more than 50 cells were counted as CFU-F colonies. For CFU-C assays, the cell suspensions were mixed with MethoCult (Stem Cell Technologies), plated in 35-mm dishes and cultured at 37°C under 5% CO_2. Colonies were scored after 14 days.

Cell differentiation

For the differentiation of three lineages, cultured cells were harvested by trypsinization, and 1×10^4 to 2×10^5 cells were transferred to an eight-well chamber and cultured overnight in culture medium. To induce osteocyte differentiation, the adherent cells were cultured in

osteogenic induction medium (LONZA, Walkersville, USA), which was changed every three to four days. After 14 days, alkaline phosphatase enzymatic staining confirmed the differentiation of cells into osteocytes. To induce adipocyte differentiation, the adherent cells were cultured in adipogenic induction medium (LONZA), which was changed every three to four days. After 14 days, Oil red O staining confirmed the differentiation of the cells into adipocytes. To induce chondrocyte differentiation, the adherent cells were cultured in a polystyrene tube containing chondrogenic induction medium (LONZA) supplemented with TGF-β3 (10 ng/ml). After 21 days, toluidine blue staining confirmed the differentiation of the cells into chondrocytes. Quantitative analysis of the differentiation capacity entailed using microscopic observation to determine the ratio of the number of differentiated cells to the number of nuclei in each field of view (100 cells).

Antibody staining and flow cytometry

Cells were suspended in ice-cold HBSS at 1 to 5×10^7 cells/ml, and were then stained for 30 minutes on ice with a monoclonal antibody. The antibodies used were LNGFR-PE, MSCA-1-APC (Miltenyi Biotec), Lineage-FITC, THY-1-FITC, THY-1-APC, SSEA-1-APC, CD34-PE, CD49f-PE, CD73-PE, CD105-PE, VCAM-1-APC, CD140b-PE, CD146-PE (BD Pharmingen, BioLegend), STRO-1-FITC (Santa Cruz Bio Technology), CD45-FITC and GPA-FITC (DAKO). Propidium iodide (PI: 2 μg/ml) was used to eliminate dead cells from the flow cytometric analysis. Flow cytometric analysis and sorting were performed on a triple-laser MoFlo (Beckman Coulter), FACSVantage SE or FACS Calibur flow cytometer (Becton Dickinson), and the data were analyzed using Flowjo software (Tree Star, Inc.). Analysis of cells populations expressing varying combinations of LNGFR and THY-1 on their surface from the different tissues routinely demonstrated 99% purity.

Homing assay

$LNGFR^+THY\text{-}1^+$ cells (freshly isolated: 1×10^4 cells, and cultured: $2\text{x}10^5$, $3\text{x}10^5$ and $1\text{x}10^6$ cells) were transplanted intravenously into the retro-orbital plexus of anesthetized recipient mice (NOG) that had been irradiated (3.5 Gy), along with 2×10^5 whole BM cells from NOG mice, as radioprotective cells. After three months, femurs from transplanted NOG mice were dissected and crushed with a pestle. Bone and marrow were then treated for one hour with 0.2% collagenase. The suspension was filtered through a cell strainer to remove debris and bone fragments and the cells were resuspended for 5 to 10 seconds in 1 ml of water to lyse the red blood cells. Transplanted mouse BM was stained for 30 minutes on ice with a monoclonal antibody (Isotype control, anti-human THY-1 and anti-human LNGFR antibody).

Senescence-associated β-galactosidase (SA-β-gal) assay

MSCs were isolated and treated as indicated in an eight-well chamber. The SA-β-gal assay was performed with a Senescence Detection Kit (BioVison, Mountain View, CA). Quantitative analysis of the senescence cells entailed using microscopic observation to determine the ratio of the number of X-gal positive cells (150-200 cells).

DNA preparation and quality assessments

A maximum of 5×10^6 cells (RECs, MECs and SECs at six weeks) were centrifuged for 5 minutes at 300 *g*. The pellet was resuspended in 200 µl PBS, and 20 µl proteinase K (Qiagen DNeasy Blood & Tissue Kit) and 4 µl RNase A (100 mg/ml) were added, mixed on a vortex mixer, and incubated for 2 min at room temperature. Then the suspension was spun in a microcentrifuge for 30 seconds at 6,000 *g* to drive the contents off the walls and lid. Genomic DNA (gDNA) was isolated using a Qiagen DNeasy Blood & Tissue Kit and Agilent's recommended procedure (http://www.chem.agilent.com/Library/usermanuals/Public/G4410-90040 CGH Bravo Protocol 1.1.pdf). The final elution volume was 400 µl in Buffer AE. The yield and purity of the gDNA were measured using a NanoDrop ND-1000 spectrophotometer (Thermo Fisher Scientific). To assess the quality and average molecular weight of the collected gDNA, aliquots from each sample were subjected to agarose gel electrophoresis. For experimentation, high-quality gDNA samples with a 260/280 ratio of 1.8 to 2.0 (the 260/230 ratio for pure DNA is >1.8) were used.

Oligonucleotide array CGH analysis

The aCGH experiments were performed according to the manufacturer's protocol (Agilent Oligonucleotide Array-Based CGH for Genomic DNA Analysis, Version 6.0, Direct Method). Briefly, 1.0 µg aliquots of sample DNA from RECs, MECs or SECs, as well as reference DNA (Promega, female, p/n G1521), were digested with *Alu*I and *Rsa*I restriction enzymes and fluorescently labeled with Cy5 (test) or Cy3 (reference) using an Agilent Genomic DNA Enzymatic Labeling Kit (p/n 5190-0449). Experimental and reference targets for each hybridization were purified using a Microcon YM-30 column (Millipore) and validated using a NanoDrop ND-1000 to ensure the yield and specific incorporation of the labeled gDNA. Labeled test and reference DNAs were combined, denatured, pre-annealed with Cot-1 DNA (Invitrogen, Carlsbad, CA) and blocking reagent (Agilent), and then hybridized to Agilent Human 4x180K CGH arrays (Design ID = 022060) for 24 hours in a rotating oven (Agilent Technologies) at 65°C and 20 rpm. After the hybridization, the array slides were washed

and then scanned at 3 μm resolution with an Agilent G2565CA Scanner. Data were extracted from the microarray images using Agilent Feature Extraction Software v10.5.1.1 with the Feature Extraction protocol CGH-105_Dec09. These ratio data, along with the associated error values and flagged features, were imported into the DNA Analytics Software v5.0.14 (Agilent). To make aberration calls, an aberration detection algorithm, ADM-2 (Lipson et al., 2006), was used with a threshold of 5.5 and an aberration filter set at 2 for the minimum number of probe regions and 0.25 for the minimum absolute average log2 ratio for regions in the DNA Analytics to reduce false positives. All aCGH data are available for viewing at the Gene Expression Omnibus (GEO) under accession number GSE34484.

Real time RT-PCR assay

VCAM-1 gene expression was analyzed via real time PCR using SYBR Premix Ex Taq (TAKARA), 100 ng of cDNA, and 200 nM primers in 25 μl reactions. Cycling parameters were: 95°C for 10 s and then 40 cycles of 95°C for 10 s and 60°C for 30 s. Generation of a single product was confirmed with a melt cycle. Real time RT-PCR was performed using an Mx 3000P (Stratagene). Primer sequences were as follows: *VCAM-1:* forward, 5-TGT TGA GAT CTC CCC TGG AC-3 and reverse, 5-CGC TCA GAG GGC TGT CTA TC-3; *human P14ARF*: forward, 5-TAC TGA GGA GCC AGC GTC TA-3 and reverse, 5'-TGC ACG GGT CGG GTG AGA GT-3; *human P16INK4a*: forward, 5-AGC ATG GAG CCT TCG GCT GA-3 and reverse, 5-CCA TCA TCA TGA CCT GGA TCG-3; *human P21*: forward, 5-GAG ACT CTC AGG GTC GAA AA-3 and reverse, 5-TTA GGG CTT CCT CTT GGA GA-3; *GAPDH:* forward, 5-TGC ACC ACC AAC TGC TTA GC-3 and reverse, 5-GGC ATG GAC TGT GGT CAT GAG-3 (Sugihara et al., 2011).

Immunochemistry

After fixation using 4% (wt/vol) paraformaldehyde, cells were incubated with 1× PBS containing 0.3% (wt/vol) TritonX-100. After washing, the cells were first incubated overnight at 4°C under rotation with anti-VCAM-1 (Abcam, 1:200), anti-Ki67 (Novo Castra, 1:200), F-actin: Alexa Fluor 488 phalloidin, (Molecular Probes, 1:1000) and a-tubulin (Sigma-Aldrich, 1:2000) antibodies diluted in Blocking One (NACALAI TESQUE, INC.), and then for one hour at room temperature with the secondary antibody. Nuclei were counterstained using Hoechst 33258 (1:1000 dilution, Sigma). For microscopy, RECs, MECs and SECs were mounted on glass slides under coverslips before analysis on a BZ-9000 microscope (KEYENCE). Quantitative analysis of the F-actin positive cells (100-200 cells, n = 3 per group) and cell width (n = 30) entailed using microscopic observation to determine the ratio and distribution.

Migration Assay

Polyethylene terephthalate (PET) filters with 8 micrometer pores (BD Biocoat) separating the upper and lower chambers, were coated with 10 μg/ml fibronectin (Sigma) for 2.5 h at 37℃. MSCs, serum-starved overnight, were trypsinized, stained with trypan blue, and counted using a hemocytometer. Before starting the migration assay, cells were treated with a blocking Ab (Isotype control [mouse IgG1], anti-VCAM-1 [51-10C9], anti-CD49d [9F10], each at 10μg/ml, BD Bioscience) for 30 min. A total of 1.5×10^4 cells were resuspended in DMEM containing 1% FBS, 10 μg/ml aphidicolin (Sigma), and were allowed to migrate towards the same culture medium supplemented with 20% FBS in the lower chamber. After 6 hours at 37℃, the filters were fixed for 20 min at room temperature with 4% paraformaldehyde in PBS (pH 7.5), and the non-migrated cells were removed by wiping the upper side of the membranes with cotton swabs. Cell nuclei were stained using Hoechst 33258, and the filters were mounted on slides in Fluoromount medium (Diagnostic Biosystems). Using Axiovision software connected to an epifluorescence microscope (Zeiss, Axioplan), 12 fields per membrane were captured, and the cells were counted manually.

Imaging of transplanted cultured MSCs *in vivo*

RECs, MECs/SECs and cultured MSCs were expanded, infected with Venus-ffLuc lentivirus (Hara-Miyauchi et al., 2012), continuously propagated, harvested, and then dissociated into single cells. The PI-negative/Venus-positive cells (1×10^5 cells) were sorted by a flow cytometry and transplanted intravenously. One-day post injection the treated mice were anesthetized and given D-luciferin (150 mg/kg body weight) i.p. We used a Xenogen-IVIS 100-cooled CCD optical macroscopic imaging system (SC BioScience Corporation) for BLI. By using the fluorescent and luminescent fusion protein, the success of lentivirus infection was confirmed with fluorescent signal from Venus. After the transplantation, the spot(s) of transplanted MSC cells were followed by bioluminescent signal from Luciferin in mice body (1×10^5 cells could be detected easily). To quantify the measured light, regions of interest (ROI) were defined as lungs and all values were examined from an equal ROI.

SUPPLEMENTAL REFERENCES

Hara-Miyauchi, C., Tsuji, O., Hanyu, A., Okada, S., Yasuda, A., Fukano, T., Akazawa, C., Nakamura, M., Imamura, T., Matsuzaki, Y.*, et al.* (2012). Bioluminescent system for dynamic imaging of cell and animal behavior. Biochemical and biophysical research communications *419*, 188-193.

Lipson, D., Aumann, Y., Ben-Dor, A., Linial, N., and Yakhini, Z. (2006). Efficient calculation of interval scores for DNA copy number data analysis. Journal of computational biology : a journal of computational molecular cell biology *13*, 215-228.

Sugihara, E., Shimizu, T., Kojima, K., Onishi, N., Kai, K., Ishizawa, J., Nagata, K., Hashimoto, N., Honda, H., Kanno, M., *et al.* (2011). Ink4a and Arf are crucial factors in the determination of the cell of origin and the therapeutic sensitivity of Myc-induced mouse lymphoid tumor. Oncogene.
